# Substituent effect on TADF properties of 2-modified 4,6-bis(3,6-di-*tert*-butyl-9-carbazolyl)-5-methylpyrimidines

**DOI:** 10.3762/bjoc.18.52

**Published:** 2022-05-05

**Authors:** Irina Fiodorova, Tomas Serevičius, Rokas Skaisgiris, Saulius Juršėnas, Sigitas Tumkevicius

**Affiliations:** 1 Institute of Chemistry, Vilnius University, Naugarduko 24, LT-03225, Vilnius, Lithuania; 2 Institute of Photonics and Nanotechnology, Vilnius University, Saulėtekio 3, LT-10257 Vilnius, Lithuania

**Keywords:** carbazole, pyrimidine, RTP, synthesis, thermally activated delayed fluorescence

## Abstract

The interest in organic materials exhibiting thermally activated delayed fluorescence (TADF) significantly increased in recent years owing to their potential application as emitters in highly efficient organic light emitting diodes (OLEDs). Simple modification of the molecular structure of TADF compounds through the selection of different electron-donating or accepting fragments opens great possibilities to tune the emission properties and rates. Here we present the synthesis of a series of novel pyrimidine–carbazole emitters and their photophysical characterization in view of effects of substituents in the pyrimidine ring on their TADF properties. We demonstrate that electron-withdrawing substituents directly connected to the pyrimidine unit have greater impact on the lowering of the energy gap between singlet and triplet states (Δ*E*_ST_) for efficient TADF as compared to those attached through a phenylene bridge. A modification of the pyrimidine unit with CN, SCH_3_, and SO_2_CH_3_ functional groups at position 2 is shown to enhance the emission yield up to 0.5 with pronounced TADF activity.

## Introduction

**Figure 1:**
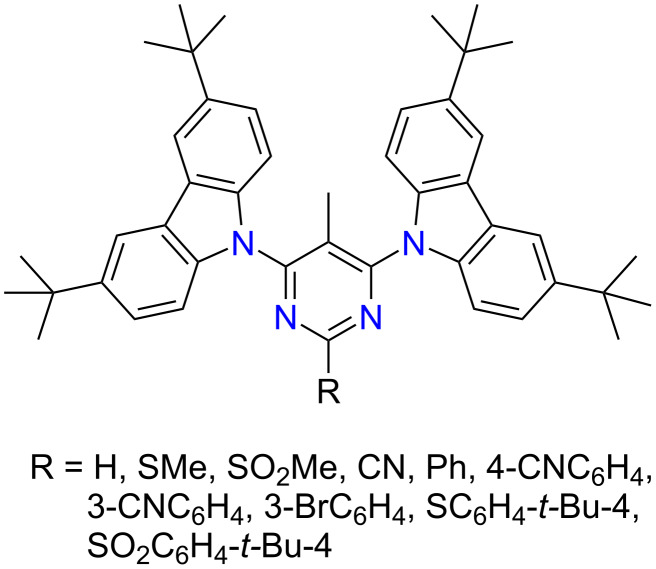
2-Modified 4,6-bis(3,6-di-*tert*-butyl-9*H*-carbazol-9-yl)-5-methylpyrimidines.

The first reports on highly efficient thermally activated delayed fluorescence (TADF) mechanism and its successful realization in organic light emitting diodes (OLEDs) by Adachi and co-workers [1–2] have drawn the attention to the design and synthesis of various emissive donor–acceptor organic compounds. Efficient triplet harvesting in TADF compounds leads to internal quantum efficiencies up to 100% and electroluminescence yields exceeding 20% [3–4]. TADF OLEDs usually contain pure organic molecules, which avoid expensive noble metals and allow broad possibilities of molecular design. One of the main requirement for efficient TADF is a negligible energy difference between the lowest singlet and triplet states (∆*E*_ST_) which is often obtained in (hetero)aromatic compounds possessing twisted electron-donor (D) and acceptor (A) fragments with strong intramolecular charge transfer (ICT) [5–7]. Moreover, the number and nature of various side units on the emitter framework can also affect the properties of TADF compounds [3]. Among the electron-donating units, 9,10-dihydroacridine, carbazole or phenoxazine derivatives often are used as D units, while the π-electron-deficient nitrogen heterocycles such as triazine, diazines or aromatics containing cyano and sulfone groups are popular acceptor units for the construction of highly efficient TADF emitters [8–11]. Pyrimidine (1,3-diazine) owing to its aromaticity, significant π-deficiency, strong electron affinity, high luminous efficiency, good electrical and optical properties, and easy chemical modification is a desired structural unit in organic structures targeted for numerous applications including organic photovoltaic solar cells (OPV) [12–14], organic field-effect transistors (OFETs) [15–17], chemical and biosensors [18–23], and OLEDs [3,7–9,24]. In case of TADF emitters, a pyrimidine ring is often connected with a donor unit through a phenylene group as a conjugate π-spacer (s) [25–28]. As the majority of research describes TADF compounds with D-s–A–s–D layout, reports on TADF properties of pyrimidine emitters where the pyrimidine moiety is directly bonded with a donor moiety are scarce [29–31], though several examples of such pyrimidine-based conjugates found utility as high triplet energy hosts [32–33]. Envisaging the potential of the pyrimidine–carbazole pair for achieving efficient deep-blue emission, we were interested to study the influence of substituents in position 2 of the pyrimidine ring on the TADF properties of pyrimidine–carbazole emitters. For this purpose, we performed the synthesis of novel 4,6-bis(3,6-di-*tert*-butyl-9*H*-carbazol-9-yl)pyrimidines modified with various substituents in position 2 of the pyrimidine ring (Figure 1).

For comparison, analysis of the TADF properties of a similar pyrimidine–carbazole emitter **tCbz-mPYR** is also included in this paper [29]. To enhance the electron-accepting character of the pyrimidine moiety some electron-withdrawing groups, namely cyano, bromo or sulfonyl groups were introduced directly or through a phenylene bridge into position 2 of the pyrimidine ring.

## Results and Discussion

### Synthesis

**Scheme 1:**
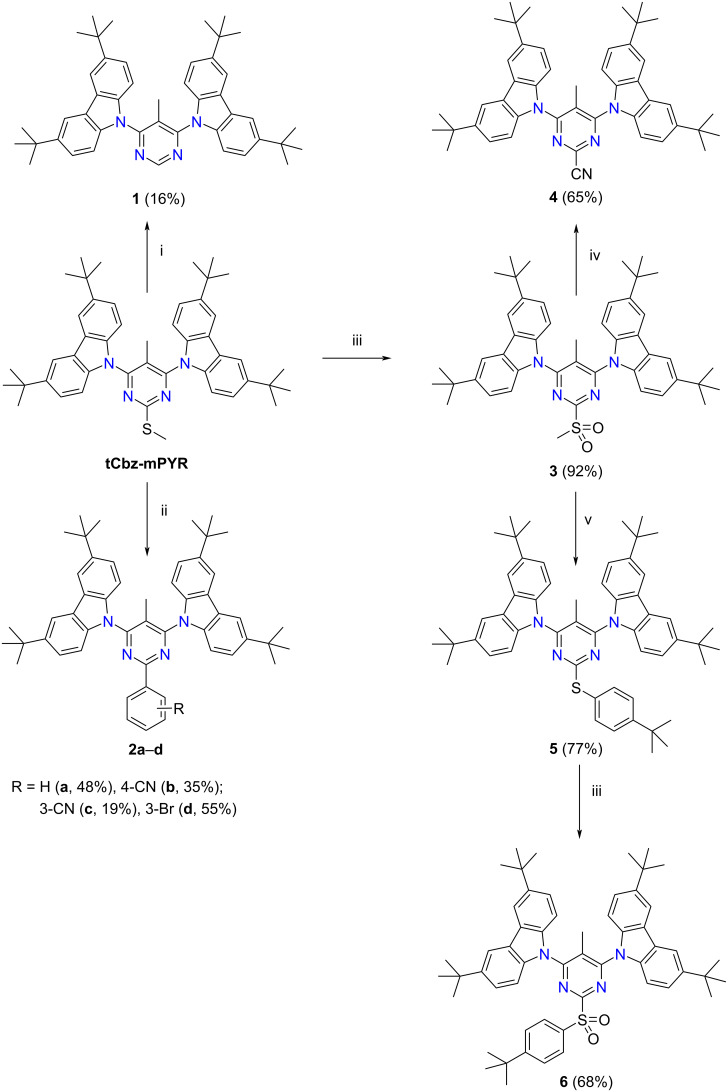
Synthesis of 4,6-bis(3,6-di-*tert*-butyl-9*H*-carbazol-9-yl)-5-methyl-2-substituted pyrimidines **1**–**6**. Reagents and conditions: i – Raney Ni, MeOH, 90 °C, 2 h; ii – arylboronic acid (1.3 equiv), Pd(PPh_3_)_4_ (5 mol %), Cs_2_CO_3_ (2 equiv; 1 equiv in case of *meta*-substituted boronic acids), copper(I) 3-methylsalicylate (2.2 equiv), dioxane, 130 °C, 4 h, argon; iii – oxone (2.5 equiv), DMF, 80 °C, 3 h; iv – NaCN (2.2 equiv), THF, reflux, 3.5 h; v – 4-*t-*BuC_6_H_4_SH (1.1 equiv), Et_3_N (1.1 equiv), THF, 50 °C, overnight, argon.

Due to the versatile reactivity of a methylthio group in the pyrimidine nucleus [34–35] we used 4,6-bis(3,6-di-*tert*-butyl-9*H*-carbazol-9-yl)-5-methyl-2-methylthiopyrimidine (**tCbz-mPYR**) as a starting material for the synthesis of 2-substituted pyrimidine emitters (Scheme 1). Compound **tCbz-mPYR** was synthesized by using the palladium-catalyzed Buchwald–Hartwig amination reaction of 4,6-dichloro-5-methyl-2-methylpyrimidine with 3,6-di-*tert*-butylcarbazole according to the procedure reported by us previously [29].

In order to remove the methylthio group in **tCbz-mPYR** and to obtain the 2-unsubstituted pyrimidine derivative **1**, a hydrogenolysis reaction employing Raney Ni was carried out. Efficient methods for the introduction of aryl moieties into methylthio-substituted nitrogen heterocycles such as **tCBz-mPYR** are a Ni(0)-catalyzed cross-coupling reaction with Grignard reagents [34,36] or the Liebeskind–Srogl reaction employing arylboronic acids [37–39]. Taking into account a large assortment of arylboronic acids and the simplicity of the method, we chose the Liebeskind–Srogl cross-coupling reaction for the synthesis of the target 2-arylpyrimidine derivatives. Thus, heating **tCbz-mPYR** with phenyl-, 4-cyanophenyl-, 3-cyanophenyl-, or 3-bromophenylboronic acid at 130 °C in dioxane in the presence of Pd(PPh_3_)_4_, copper(I) 3-methylsalicylate (CuMeSal), and Cs_2_CO_3_ as a catalyst system furnished the corresponding 2-substituted pyrimidines **2a**–**d**. For the introduction of cyano and 4*-*(*tert*-butyl)phenylthio groups into position 2 of the pyrimidine, the conversion of the methylthio group to the better leaving methylsulfonyl group was necessary to perform. A suitable oxidant for this purpose appeared to be oxone [40]. Thus, the oxidation of **tCbz-mPYR** with oxone proceeded in DMF at 80 °C to provide the 2-methylsulfonyl derivative **3** in 92% yield. Then, treatment of compound **3** with NaCN or 4-(*tert*-butyl)thiophenol led to the formation of the 2-cyano- and 2-(4-*tert*-butylphenylthio) derivatives **4** and **5** in 65% and 77% yield, respectively. Finally, compound **5** in the reaction with oxone furnished 2-(4-*tert*-butylphenylsulfonyl) derivative **6** in reasonable 68% yield. ^1^H and ^13^C NMR spectroscopy and HRMS were employed to confirm the structures of the synthesized compounds (see Figures S1–S27 in Supporting Information File 1).

### DFT analysis

**Figure 2:**
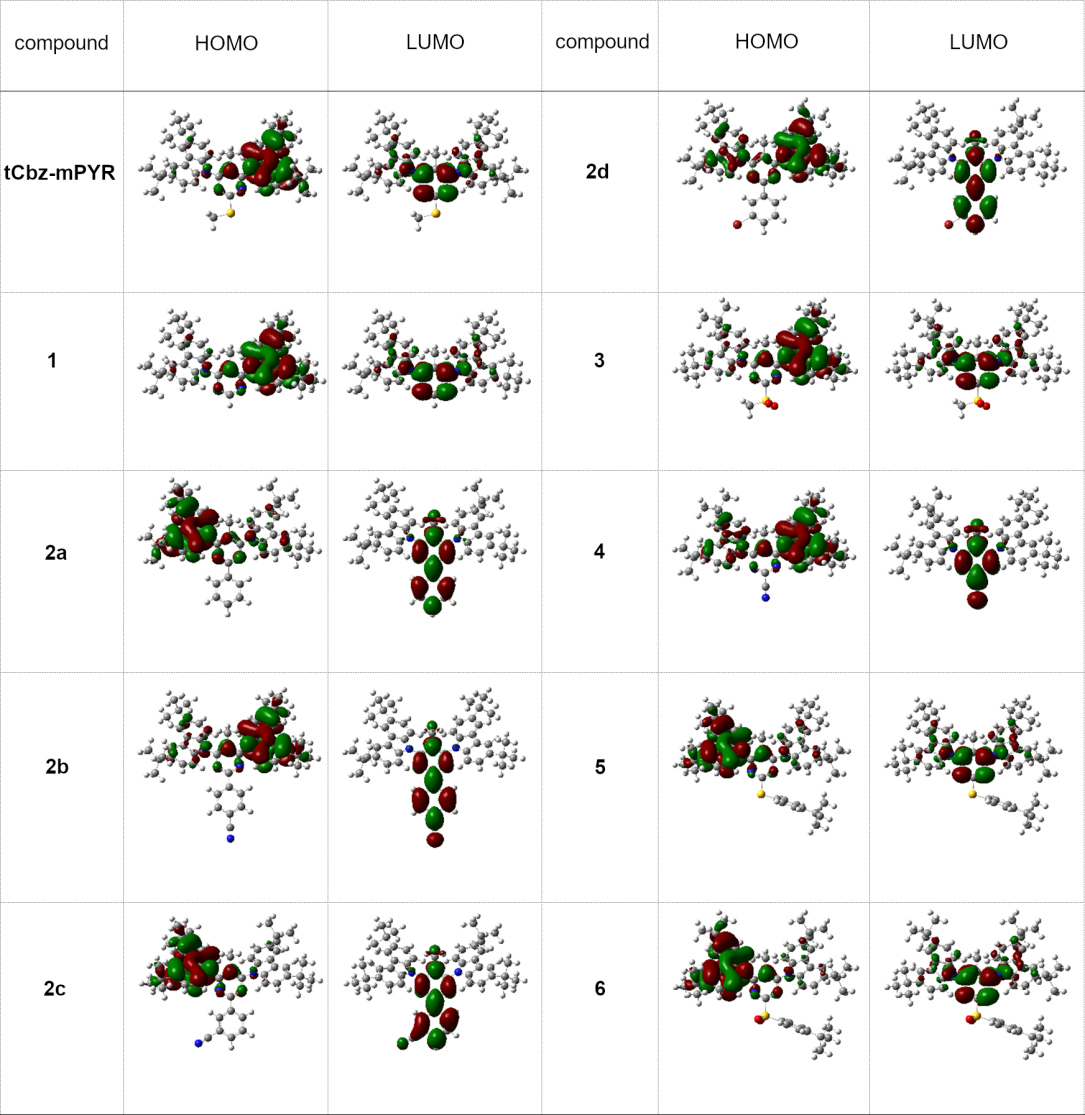
HOMO and LUMO spatial distributions of carbazole–pyrimidine TADF compounds.

To assess the structural and electronic properties of the chromophores **tCbz-mPYR** and **1**–**6**, quantum chemical calculations were performed. DFT analysis revealed that all studied compounds were of similar molecular geometry with partially twisted carbazole units (in the order of 45–47°). The steric hindrance between the carbazole fragments and the pyrimidine core was enhanced by introducing a methyl group in position 5 of the latter [29], enabling sufficient HOMO–LUMO decoupling. Single-bonded phenyl substituents (compounds **2a**–**d**) are almost coplanar with the pyrimidine core (dihedral angle 0.5–1.5°), while a phenyl group connected with the pyrimidine ring via a sulfur atom (compounds **5** and **6**) is twisted out of the pyrimidine plane and is not conjugated with the pyrimidine ring. Despite the similar molecular structures, the studied chromophores showed somewhat different electronic properties, mainly due to the variation of the acceptor structure. The DFT computed spatial distribution of frontier molecular orbitals (FMOs) of compounds **tCbz-mPYR** and **1**–**6** is presented in Figure 2. A comparison of the electronic structures revealed that the electron density distribution in the HOMO is rather similar for all molecules and tended to localize on the electron-donating carbazole moiety partially extending to the pyrimidine ring. The main differences in the electronic structure can be observed in the LUMO distribution. For the 2-methylthio- and 2-(*tert*-butylphenyl)thio-substituted compounds (**tCbz-mPYR** and **5**) or the corresponding sulfonyl derivatives (**3** and **6**), the LUMO is localized over the electron-withdrawing pyrimidine unit with low extension to tCbz, being very similar to the LUMO distribution for pyrimidine derivative **1** without a substituent at the position 2 of the pyrimidine ring. The π-electron density distribution in the LUMO of compounds bearing a phenyl (**2a**), *para/meta*-cyanophenyl, and *meta*-bromophenyl moiety (**2b**–**d**) at position 2 of the pyrimidine ring tends to localize over the pyrimidine ring and nearby phenyl groups, resulting in a lower HOMO–LUMO overlap. A similar LUMO localization is observed for compound **4** with a 2-cyano group.

The energies of the HOMO level were in the range from −5.50 eV to −5.70 eV with lower values for those compounds bearing electron-withdrawing groups (Table 1). The LUMO energies varied from −1,38 eV for compound **5** with a phenylthio group to −2.15 eV for compound **2b** bearing a *p*-cyanophenyl group in position 2 of the pyrimidine ring. The S_0_→S_1_ transition energies ranged from 3–3.4 eV with lower values for the CN/Br/phenyl-substituted compounds due to the larger conjugation length of the acceptor unit (also evidenced by a deeper LUMO), followed by lower oscillator strengths of S_0_→S_1_ transition due to more evident HOMO–LUMO decoupling (≈0.04 vs ≈0.4). Lower singlet–triplet energy gaps were estimated for compounds with stronger acceptor units (down to 200 meV), beneficial for efficient thermal triplet upconversion.

**Table 1:** DFT computed S_0_→S_1_/T_1_ transition energies, oscillator strengths of S_0_→S_1_ transitions, and Δ*E*_ST_ values of carbazole–pyrimidine TADF compounds.

Compd.	*f*_S0→S1_^a^	*E*_S0→S1_ (eV)^b^	*E*_S0→T1_ (eV)^c^	Δ*E*_S1T1_ (meV)^d^	HOMO (eV)^e^	LUMO (eV)^f^

**tCbz-mPYR**	0.3940	3.4265	3.1380	289	−5.53	−1.42
**1**	0.3999	3.4508	3.1400	311	−5.53	−1.39
**2a**	0.0383	3.4094	2.9911	418	−5.51	−1.52
**2b**	0.0360	2.9562	2.7535	203	−5.61	−2.15
**2c**	0.0473	3.1616	2.9003	261	−5.57	−1.89
**2d**	0.0465	3.2820	2.9518	330	−5.55	−1.71
**3**	0.3959	3.2132	2.9251	288	−5.66	−1.79
**4**	0.0496	3.0245	2.7858	239	−5.70	−2.09
**5**	0.4100	3.4377	3.1390	299	−5.50	−1.38
**6**	0.3779	3.2706	2.9864	284	−5.63	−1.71

^a^S_0_→S_1_ transition oscillator strength; ^b^S_0_→S_1_ transition energies; ^c^S_0_→T_1_ transition energies; ^d^singlet–triplet energy gap; ^e^HOMO energies; ^f^LUMO energies.

### Absorption and emission properties

**Figure 3:**
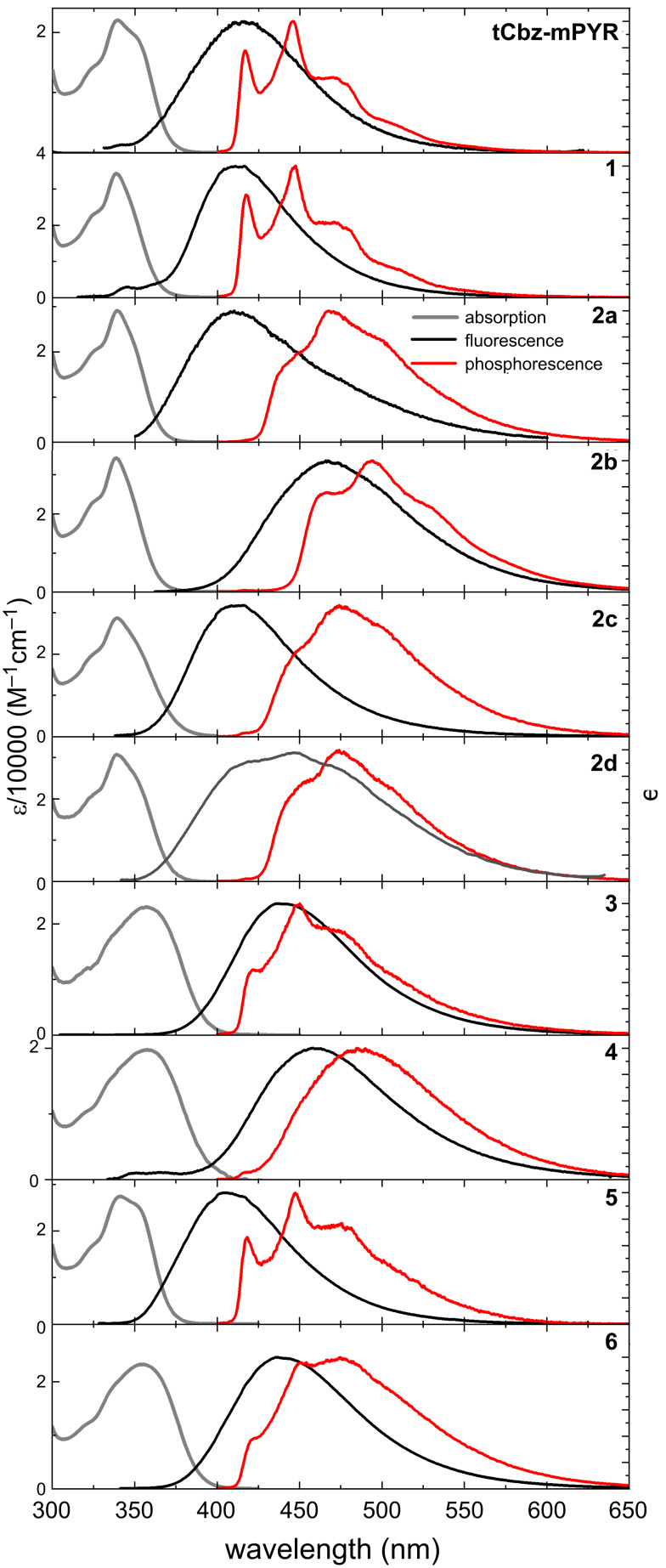
Absorption (grey lines), fluorescence (black lines) and 10K phosphorescence (red lines) spectra of carbazole–pyrimidine TADF compounds. 10^−5^ M toluene solutions were used for absorption measurements, while 1 wt % PMMA films were used for emission measurements.

Absorption (10^−5^ M toluene solutions) and emission spectra (1 wt % PMMA films) of the carbazole–pyrimidine TADF compounds are shown in Figure 3 and details are enclosed in Table 2. The absorption spectra were rather similar for all compounds, described with, typical for tCbz units, vibronic progression-having peaks bellow ≈340 nm [41]. Molar absorption coefficients were in the range of 19700–34200 M^−1^ cm^−1^, being in line with DFT predicted S_0_→S_1_ oscillator strengths and LUMO energies. The fluorescence spectra, on the other hand, showed much larger differences upon the modification of the acceptor unit. In this case, the emission wavelength was tuned in the range of 480 meV, starting from 411 nm (compounds **1** and **2a**) to 468 nm (compound **2b**). Such trend was in line with the increase of the strength of the acceptor unit, namely the energy of the LUMO (see Table 1 and Figure S28 in Supporting Information File 1), while the lower emission energy was observed for compounds with more pronounced charge-transfer (CT) character. This was also evidenced by larger FWHM values of fluorescence spectra for compounds with stronger CT emission (e.g., compounds **1** and **4**). The phosphorescence (PH) spectra of the carbazole–pyrimidine TADF compounds were rather intriguing. 10 K PH spectra of compounds **tCbz-mPYR**, **1**, **3**, **5**, and **6** clearly resembled that of individual tCbz units, peaking at about 418 nm [29], while for the rest of the compounds the PH spectra were red-shifted, though still maintaining the vibronic structure. However, the closer inspection of the PH spectra, see Figure S29 in Supporting Information File 1, revealed that all PH spectra coincided with that of the tCbz unit. Actually, the lowest-energy 0–0’ vibronic peak was of very low intensity for compounds **2a**–**d** or **4** as well as the intensity ratio of high-energy replicas was also different, though the T_1_ energy was the same for all carbazole–pyrimidine compounds. The compounds **2a**–**d** and **4** have one common feature, namely, their acceptor unit is modified with various phenyl-like fragments, increasing the flexibility of the molecular structure, probably altering the electron–vibronic coupling. A similar behavior was observed for similar pyrimidine TADF compounds [42]. Moreover, fluorescence line shapes of compounds **2a** and **2d** were different from the rest of compounds with low-energy shoulder. More detailed analysis (see Figure S30 in Supporting Information File 1) revealed the presence of room-temperature phosphorescence (RTP), perturbing the lineshape of PL spectra. The estimated singlet–triplet energy gaps (Δ*E*_ST_) of carbazole–pyrimidine TADF compounds were in the range of 159–530 meV, systematically decreasing for compounds with stronger acceptor unit (see Figure S28d in Supporting Information File 1). Δ*E*_ST_ was large for the majority of compounds, however, for compounds **2b, 3**, **4**, and **6** modified with CN and SO_2_CH_3_ groups Δ*E*_ST_ was as low as 159 meV, promising for efficient thermally activated triplet upconversion.

**Table 2:** Fluorescence parameters of carbazole–pyrimidine TADF compounds.

Compd.	λ_abs_ (nm)^a^	ε (M^−1^cm^−1^)^b^	λ_PL_ (nm)^c^	Φ_PF_^d^	Φ_DF_^e^	*k*_PF_ (×10^8^ s)^f^	*k*_r_ (×10^6^ s)^g^	*k*_nr_ (×10^8^ s)^h^	Φ_DF_/Φ_PF_^i^	Δ*E*_ST_ (meV)^j^

**tCbz-mPYR**	339	22000	415	0.05	0.40	2.5	13.0	2.4	8.0	530
**1**	339	33420	411	0.07	0.11	1.4	9.3	1.3	1.6	397
**2a**	339	29100	411	0.03	0.06	2.1	6.8	2.0	2.0	510
**2b**	339	34200	468	0.01	0.24	3.1	3.0	3.1	24.0	128
**2c**	339	28700	410	0.01	0.18	3.0	3.7	3.0	18.0	443
**2d**	339	30700	447	0.01	0.05	4.2	2.4	4.1	5.0	501
**3**	357	23000	440	0.06	0.49	0.7	4.0	0.6	8.2	265
**4**	358	19700	460	0.01	0.34	1.1	1.3	1.0	34.0	159
**5**	341	27400	408	0.03	0.05	3.0	8.2	2.9	1.7	530
**6**	355	23000	441	0.04	0.02	1.2	5.1	1.1	0.5	273

^a^Absorption peak in toluene; ^b^molar absorption coefficient in toluene; ^c^fluorescence peak in 1 wt % PMMA; ^d^prompt fluorescence quantum yield 1 wt % PMMA; ^e^delayed fluorescence quantum yield 1 wt % PMMA; ^f^fluorescence decay rates 1 wt % PMMA; ^g^radiative decay rates 1 wt % PMMA; ^h^nonradiative decay rates 1 wt % PMMA; ^i^prompt and delayed fluorescence quantum yield ratio 1 wt % PMMA; ^j^singlet–triplet energy gap 1 wt % PMMA.

**Figure 4:**
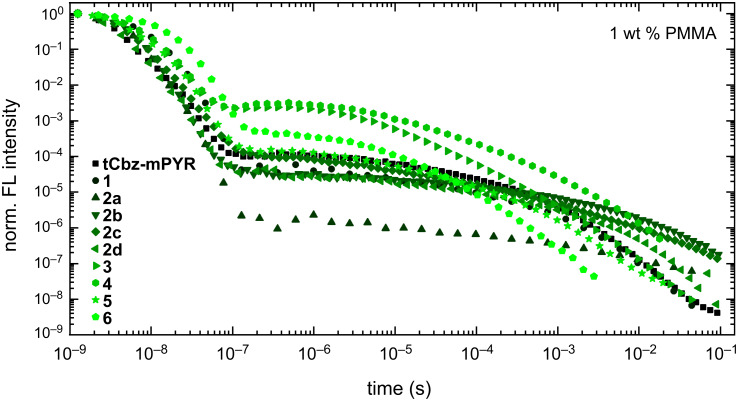
Fluorescence decay transients of 1 wt % PMMA films of carbazole–pyrimidine TADF compounds in oxygen-free conditions.

Fluorescence decay transients of 1 wt % PMMA films of carbazole–pyrimidine TADF compounds are shown in Figure 4. Typical for TADF compounds temporal profiles having two decay regimes were observed when the initial decay was of prompt fluorescence and the latter one was of TADF. The PF decay rate (1–4 × 10^8^ s^−1^) was typical for directly bound carbazole–pyrimidine TADF compounds [43–44], though a somewhat lower value was estimated for compound **3** modified with a methylsulfonyl group. More insights could be drawn by analyzing the fluorescence quantum yield (Φ_F_) as well as radiative (*k*_r_) and non-radiative (*k*_nr_) fluorescence decay rates (see Table 2). The Φ_PF_ was typical for carbazole–pyrimidine TADF compounds [31,43–44], in the range of 0.01–0.07 and decreased for compounds with stronger acceptor units (see Figure S28b in Supporting Information File 1). The radiative decay rate was in the range of 1.3–13.0 × 10^6^ s^−1^. Typically, *k*_r_ was larger for compounds with weaker acceptor units and a lower CT fluorescence strength (see Figure S28c in Supporting Information File 1). In case of *k*_nr_, a similar analysis is hardly possible, as *k*_nr_ accounts for two different rates, namely internal conversion to S_0_ and intersystem crossing. However, some faint trends could be seen, as compounds with the strongest donor units tend to have the larger *k*_nr_ (compounds **2b**–**d**).

In case of delayed fluorescence, more complicated fluorescence temporal profiles were observed, aggravating its analysis. DF decay transients were perturbed by conformational disorder, typical for D–A TADF compounds in solid films [41,45–47]. The presence of conformational disorder made exponential fitting of DF transients hardly possible due to the multiexponential temporal profiles of the DF decay. Unfortunately, TADF decay rates were inaccessible. Similar emission decay transients were also estimated for RTP compounds **2a** and **2d** both at singlet and triplet emission peaks (see Figure S31 in Supporting Information File 1). The largest DF quantum yield, ranging from 0.34–0.49, was estimated for compounds **tCbz**-**mPYR**, **3**, and **4** with the lowest Δ*E*_ST_, modified with CN, SCH_3_, and SO_2_CH_3_ functional groups directly at the pyrimidine unit. The introduction of phenyl substituents (compounds **2a**–**d**) or S-aryl units (compounds **5** and **6**) at position 2 of the pyrimidine ring led to lower Φ_DF_, probably due to the enlarged nonradiative decay rate. Interestingly, Δ*E*_ST_ was remarkably larger for **tCbz**-**mPYR** despite its high Φ_DF_. In this case, rISC probably was promoted by efficient triplet upconversion through spin-vibronicaly bound T_n_ energy levels [48].

## Conclusion

In summary, a series of novel pyrimidine–carbazole TADF emitters bearing different substituents in position 2 of the pyrimidine moiety were successfully prepared by using Liebeskind–Srogl cross-coupling, hydrogenolysis, oxidation reactions of 4,6-bis(3,6-di-*tert*-butyl-9-carbazolyl)-5-methyl-2-methylthiopyrimidine and following nucleophilic substitution of the methylsulfonyl group with sodium cyanide and 4-(*tert*-butyl)thiophenol. The thorough photophysical analysis was carried out to assess the impact of different substituents in position 2 of the pyrimidine ring. It was shown that HOMO–LUMO overlap and the resulting Δ*E*_ST_ can be easily minimized, enabling rather efficient TADF. We have shown that electron-withdrawing substituents connected directly to the pyrimidine unit have a larger impact on TADF efficiency in comparison with those attached through a phenylene bridge. The largest delayed fluorescence quantum yield, ranging from 0.34–0.49, was estimated for compounds with CN, SCH_3_, and SO_2_CH_3_ functional groups at the position 2 of the pyrimidine unit. We believe that our findings on the TADF properties of differently substituted carbazole–pyrimidines will be useful for molecular design of high-performance TADF emitters in the future.

## Experimental

### General information

Reagents and solvents were purchased directly from commercial suppliers and solvents were purified by known procedures. Melting points were determined in open capillaries with a digital melting point IA9100 series apparatus (ThermoFischer Scientific) and were not corrected. Thin-layer chromatography was performed using TLC aluminum sheets with silica gel (Merck 60 F254). Visualization was accomplished by UV light. Column chromatography was performed using silica gel 60 (0.040–0.063 mm, Merck). NMR spectra were recorded on a Bruker Ascend 400 (400 MHz and 100 MHz for ^1^H and ^13^C, respectively). ^1^H NMR and ^13^C NMR spectra were referenced to residual solvent peaks. High-resolution mass spectrometry (HRMS) analyses were carried out on a Dual-ESI Q-TOF 6520 (Agilent Technologies) mass spectrometer.

Photophysical properties were analyzed in 10^−5^ M toluene solutions as well as 1 wt % PMMA (poly(methyl methacrylate)) films. The solid-state samples were prepared by dissolving the compounds and polymer or host material at appropriate ratios in toluene solution and then wet-casting the solutions on quartz substrates. The absorption spectra were recorded by a Lambda 950 UV–vis–NIR spectrometer (Perkin Elmer). The fluorescence quantum yields (Φ_F_) in ambient air were estimated by the integrating sphere method [49] in integrating sphere (Sphere Optics) coupled to a CCD spectrometer PMA-12 (Hamamatsu) via optical fibers excited with a CW xenon lamp. Φ_PF_ and Φ_DF_ values were estimated according to [50]. Time-integrated fluorescence, phosphorescence spectra as well as fluorescence decay kinetics were recorded with a time-gated intensified iCCD camera iStar DH340T (Andor) with a spectrograph SR-303i (Shamrock) coupled with nanosecond YAG:Nd^3+^ laser NT 242 with an optical parametric generator (Ekspla, pulse width 7 ns, 1 kHz frequency, 100 nJ pulse energy). Fluorescence decay transients were obtained by exponentially increasing the delay and integration time [51]. Phosphorescence spectra were recorded at 10 K temperature after a 100 μs delay with a 49 ms integration time. Solid-state samples were mounted in a closed cycle He cryostat (Cryo Industries 204 N) for PL measurements in oxygen-free conditions.

Quantum chemical calculations were carried out by using density functional theory at the B3LYP/6-31G(d) level as implemented in a software package Gaussian 09 [52]. The solvation behaviour of the surrounding toluene was estimated by the polarizable continuum model. Although the B3LYP/6-31G(d) theory level due to the neglected long-range exchange interaction can give less accurate results for calculated molecular geometries and transition energies [53], however, it should be sufficient for brief analysis.

### Procedures and product characterization

**4,6-Bis[3,6-di(*tert*-butyl)-9*H*-carbazol-9-yl]-5-methylpyrimidine (1).** Compound **1** was synthesized in a manner similar to [30]. A mixture of compound **tCbz-mPYR** (90 mg, 0.13 mmol, 1 equiv), Raney nickel (270 mg, 3 equiv by mass), prepared before reaction according to [54], and methanol (3 mL) were placed in a screw-cap vial equipped with a magnetic stirring bar. The reaction mixture was heated at 90 °C under stirring for 2 h. Then, Raney nickel was filtered off and washed with hot chloroform. The filtrate was concentrated and the obtained residue was purified by column chromatography using chloroform/petroleum ether 2:1 to pure chloroform as an eluent to give 13.5 mg (16%) of compound **1**. Mp > 300 °C (from 2-propanol); ^1^H NMR (400 MHz, CDCl_3_) δ 1.52 (s, 36H, (CH_3_)_3_C), 2.01 (s, 3H, CH_3_), 7.48 (d, *J* = 8 Hz, 4H, CH_Cbz_), 7.62 (d, *J* = 8 Hz, 4H, CH_Cbz_), 8.18 (s, 4H, CH_Cbz_), 9.23 (s, 1H, CH_py_) ppm; ^13^C NMR (100 MHz, CDCl_3_) δ 15.2, 31.9, 34.9, 110.7, 116.6, 121.8, 124.2, 124.7, 137.8, 144.6, 157.1, 160.2 ppm; HRMS–ESI (*m/z*): [M + H]^+^ calcd for C_45_H_53_N_4_, 649.4265; found, 649.4265.

#### 2-Aryl-4,6-bis[3,6-di(*tert*-butyl)-9*H*-carbazol-9-yl]-5-methylpyrimidines **2a–d**

**General procedure.** 4,6-Bis(3,6-di-*tert*-butyl-carbazol-9-yl)-5-methyl-2-methylthiopyrimidine (50 mg, 0.072 mmol), the corresponding boronic acid (0.094 mmol), CuMeSal (34 mg, 0.158 mmol), Pd(PPh_3_)_4_ (4.2 mg, 0.0036 mmol), Cs_2_CO_3_ (46.8 mg, 0.144 mmol, in case of *meta-*substituted boronic acid, 1 equiv) and dioxane (3 mL) were placed in a screw-cap vial equipped with a magnetic stirring bar and flushed with argon for 10 min. The reaction mixture was heated at 130 °C under stirring for 4 h. Then, dioxane was removed by distillation under reduced pressure, water (40 mL) was added to the residue, and the mixture was extracted with chloroform (3 × 25 mL). The combined extracts were washed with brine, dried with anhydrous Na_2_SO_4_, filtered, and chloroform was removed by distillation under reduced pressure. The residue was purified by column chromatography using chloroform/petroleum ether (1:1 for compound **2a**; 1:2 for compounds **2b**, **2c**; 1:4 for compound **2d**) as an eluent to give the corresponding product.

**4,6-Bis[3,6-di(*tert*-butyl)-9*H*-carbazol-9-yl]-5-methyl-2-phenylpyrimidine (2a).** Yield 48%; mp > 320 °C (from 2-propanol); ^1^H NMR (400 MHz, CDCl_3_) δ 1.54 (s, 36H, (CH_3_)_3_C), 1.96 (s, 3H, CH_3_), 7.52–7.59 (m, 7H, ArH, CH_Cbz_) 7.61–7.66 (m, 4H, CH_Cbz_), 8.20 (s, 4H, CH_Cbz_), 8.58–8.63 (m, 2H, ArH) ppm; ^13^C NMR (100 MHz, CDCl_3_) δ 15.2, 32.0, 34.86; 111.0, 116.5, 118.4, 124.0, 124.6, 128.4, 128.7, 131.1, 136.9, 137.9, 144.4, 160.2, 163.0 ppm; HRMS–ESI (*m/z*): [M + H]^+^ calcd for C_51_H_57_N_4_, 725.4578; found, 725.4576.

**4,6-Bis[3,6-di(*tert*-butyl)-9*H*-carbazol-9-yl]-2-(4-cyanophenyl)-5-methylpyrimidine (2b).** Yield 35%; mp 266–270 °C (from 2-propanol); ^1^H NMR (400 MHz, CDCl_3_) δ 1.54 (s, 36H, (CH_3_)_3_C), 2.00 (s, 3H, CH_3_), 7.54 (d, ^3^*J* = 8.61 Hz, 4H, CH_Cbz_), 7.64 (dd, ^3^*J* = 8.61 Hz, ^4^*J* = 1.69 Hz, 4H, CH_Cbz_), 7.82 (d, ^3^*J* = 8.35 Hz, 2H, ArH), 8.21 (d, ^4^*J* = 1.61 Hz, 4H, CH_Cbz_), 8.70 (d, ^3^*J* = 8.35 Hz, 2H, ArH) ppm; ^13^C NMR (100 MHz, CDCl_3_) δ 15.4, 32.0, 34.9, 110.9, 114.3, 116.7, 118.9, 119.7, 124.1, 124.7, 128.8, 132.6, 137.7, 140.9, 144.7, 160.5 ppm; HRMS–ESI (*m/z*): [M + H]^+^ calcd for C_52_H_56_N_5_), 750.4530; found, 750.4534.

**4,6-Bis[3,6-di(*tert*-butyl)-9*H*-carbazol-9-yl]-2-(3-cyanophenyl)-5-methylpyrimidine (2c).** Yield 19%; mp 233–236 °C (from 2-propanol); ^1^H NMR (400 MHz, CDCl_3_) δ 1.44 (s, 36H, (CH_3_)_3_C), 1.89 (s, 3H; CH_3_), 7.43 (d, ^3^*J* = 8.63 Hz, 4H, CH_Cbz_), 7.52–7.56 (m, 5H, CH_Cbz_, ArH), 7.71 (d, ^3^*J* = 7.7 Hz, 1H, ArH), 8.10 (d, ^4^*J* = 1.68 Hz, 4H, CH_Cbz_), 8.72 (d, ^3^*J* = 8.0 Hz, 1H, ArH), 8.77 (s, 1H, ArH) ppm; ^13^C NMR (100 MHz, CDCl_3_) δ 15.4, 32.0, 34.9, 110.8, 113.0, 116.7, 118.7, 119.7, 124.2, 124.7, 129.6, 131.9, 132.5, 134.2, 137.7, 138.1, 144.7, 160.5, 160.8 ppm; HRMS–ESI (*m/z*): [M + H]^+^ calcd for C_52_H_56_N_5_, 750.4530; found, 750.4524.

**4,6-Bis[3,6-di(*tert*-butyl)-9*H*-carbazol-9-yl]-2-(3-bromophenyl)-5-methylpyrimidine (2d).** Yield 55%; mp > 250 °C; ^1^H NMR (400 MHz, CDCl_3_) δ 1.54 (s, 36H, (CH_3_)_3_C), 1.95 (s, 3H, CH_3_), 7.40 (t, ^3^*J* = 7.88 Hz, 1H, ArH), 7.53 (d, ^3^*J* = 8.6 Hz, 4H, CH_Cbz_), 7.63 (dd, ^3^*J* = 8.68 Hz, ^4^*J* = 1.92 Hz, 4H, CH_Cbz_), 7.67 (m, 1H, ArH), 8.20 (d, ^4^*J* = 1.64 Hz, 4H, CH_Cbz_), 8.53 (dt, ^3^*J* = 8.0 Hz, ^4^*J* = 1.24 Hz, 1H, ArH), 8.72 (t, ^4^*J* = 1.72 Hz, 1H, ArH) ppm; ^13^C NMR (100 MHz, CDCl_3_) δ 15.2, 32.0, 34.9, 110.9, 116.6, 119.2, 123.0, 124.1, 124.6, 127.0, 130.3, 131.2, 134.0, 137.8, 139.0, 144.5, 160.4, 161.7 ppm; HRMS–ESI (*m/z*): [M + H]^+^ calcd for C_51_H_56_BrN_4_, 803.3683; found, 803.3675.

**4,6-Bis(3,6-di-*tert*-butyl-9*H*-carbazol-9-yl)-5-methyl-2-methylsulfonylpyrimidine (3).** A mixture of compound **tCbz-mPYR** (60 mg, 0.086 mmol), oxone (133.2 mg, 0.217 mmol, 2.5 equiv by active component), and DMF (3 mL) were stirred at 80 °C for 3 h. After completion of the reaction, water (40 mL) was added to reaction mixture and the aqueous solution was extracted with chloroform (3 × 25 mL). The combined extract was washed with brine twice, dried with anhydrous Na_2_SO_4_, filtered, and chloroform was removed by distillation under reduced pressure. The residue was purified by column chromatography using chloroform/petroleum ether 2:1 as an eluent to give 58 mg (92%) of compound **3**. Mp > 350 °C (from 2-propanol); ^1^H NMR (400 MHz, CDCl_3_) δ 1.52 (s, 36H, (CH_3_)_3_C), 2.03 (s, 3H, CH_3_), 3.45 (s, 3H, CH_3_SO_2_), 7.54 (d, *J* = 8.65 Hz, 4H, CH), 7.64 (dd, *J* = 8.67 Hz, *J* = 1.89 Hz, 4H, CH), 8.15 (d, J = 1.68 Hz, 4H, CH) ppm; ^13^C NMR (100 MHz, CDCl_3_) δ 16.5, 31.9, 34.9, 39.4, 111.2, 116.7, 122.6, 124.4, 125.2, 137.3, 145.6, 160.8, 162.5, 163.8 ppm; HRMS–ESI (*m/z*): [M + H]^+^ calcd for C_46_H_55_N_4_O_2_S, 727.4040; found, 727.4034.

**4,6-Bis(3,6-di-*tert*-butyl-9*H*-carbazol-9-yl)-5-methylpyrimidine-2-carbonitrile (4).** A mixture of compound **3** (50 mg, 0.069 mmol), NaCN (7.4 mg, 0.151 mmol), and THF (2 mL) was refluxed under stirring for 3.5 h. After completion of the reaction, THF was removed by distillation under reduced pressure, water (30 mL) was added to the residue, and the aqueous solution was extracted with chloroform (3 × 25 mL). The combined extract was washed with brine, dried with anhydrous Na_2_SO_4_, filtered, and chloroform was removed by distillation under reduced pressure. The residue was purified by column chromatography using chloroform/petroleum ether 1:2 as an eluent to give 30 mg (65%) of compound **4**. Mp > 320 °C (from 2-propanol); ^1^H NMR (400 MHz, CDCl_3_) δ 1.43 (s, 36H, (CH_3_)_3_C), 1.92 (s, 3H, CH_3_), 7.40 (d, ^3^*J* = 8.63 Hz, 4H, CH_Cbz_), 7.55 (dd, ^3^*J* = 8.66 Hz, ^4^*J* = 1.93 Hz, 4H, CH_Cbz_), 8.07 (d, ^4^*J* = 1.67 Hz, 4H, CH_Cbz_) ppm; ^13^C NMR (100 MHz, CDCl_3_) δ 16.4, 31.9, 34.9, 111.0, 115.4, 116.8, 123.8, 124.3, 125.1, 137.2, 142.4, 145.6, 160.7 ppm; HRMS–ESI (*m/z*): [M + H]^+^ calcd for C_46_H_52_N_5_, 674.4217; found, 674,4213.

**4,6-Bis[3,6-di(*tert*-butyl)-9*H*-carbazol-9-yl]-2-[4-(*tert*-butyl)phenylthio]-5-methylpyrimidine (5).** A mixture of compound **3** (40 mg, 0.055 mmol), 4-(*tert*-butyl)thiophenol (10.4 μL, 0.061 mmol), NEt_3_ (8.4 μL, 0.061 mmol), and THF (2 mL) were placed in a screw-cap vial equipped with a magnetic stirring bar and flushed with argon for 10 min. The reaction mixture was stirred at 50 °C overnight. Then, THF was removed by distillation under reduced pressure, water (30 mL) was added to residue, and the aqueous solution was extracted with chloroform (3 × 25 mL). The combined extract was washed with brine, dried with anhydrous Na_2_SO_4_, filtered, and chloroform was removed by distillation under reduced pressure. The residue was purified by column chromatography using chloroform/petroleum ether 1:2 as an eluent to give 35 mg (77%) of compound **5**. Mp 214–217 °C (from 2-propanol); ^1^H NMR (400 MHz, CDCl_3_) δ 1.19 (s, 9H, (CH_3_)_3_C_benz_), 1.37 (s, 36H, (CH_3_)_3_C_Cbz_), 1.69 (s, 3H, CH_3_), 7.28–7.35 (m, 6H, ArH, CH_Cbz_), 7.44 (dd, ^3^*J* = 8.65 Hz, ^4^*J* = 1.66 Hz, 4H, CH_Cbz_), 7.60 (d, ^3^*J* = 8.27 Hz, 2H, ArH), 8.00 (d, ^4^*J* = 1.39 Hz, 4H, CH_Cbz_) ppm; ^13^C NMR (100 MHz, CDCl_3_) δ 15.3, 31.3, 32.0, 34.7, 34.8, 111.4, 114.8, 116.3, 123.8, 124.6, 125.6, 126.2, 135.7, 137.6, 144.5, 152.6, 159.9, 170.9 ppm; HRMS–ESI (*m/z*): [M + H]^+^ calcd for C_55_H_65_N_4_S, 813.4924; found, 813.4919.

**4,6-Bis[3,6-di(*tert*-butyl)-9*H*-carbazol-9-yl]-2-[4-(*tert*-butyl)phenylsulfonyl]-5-methylpyrimidine (6).** Compound **6** was synthesized from compound **5** according to the procedure described for compound **3**. Yield 68%, mp 275–278 °C (from 2-propanol); ^1^H NMR (400 MHz, CDCl_3_) δ 1.38 (s, 9H, (CH_3_)_3_C_benz_), 1.51 (s, 36H, (CH_3_)_3_C_Cbz_), 1.91 (s, 3H, CH_3_), 7.43 (d, ^3^*J* = 8.66 Hz, 4H, CH_Cbz_), 7.56–7.65 (m, 6H, ArH, CH_Cbz_), 8.13 (d, ^4^*J* = 1.69 Hz, 4H, CH_Cbz_), 8.17 (m, 2H, ArH) ppm; ^13^C NMR (100 MHz, CDCl_3_) δ 16.6, 31.1, 31.9, 34.9, 35.4, 111.4, 116.6, 121.3, 124.2, 125.1, 126.2, 129.9, 134.5, 137.3, 145.4, 158.2, 160.5, 164.5 ppm; HRMS–ESI (*m/z*): [M + H]^+^ calcd for C_55_H_65_N_4_O_2_S, 845.4823; found, 845.4812.

## Supporting Information

File 1 Copies of NMR spectra and extended photophysical properties.
