# Supplementary material for: Substituent effect on TADF properties of 2-modified 4,6-bis(3,6-di-tert-butyl-9-carbazolyl)-5-methylpyrimidines
Source: Beilstein J Org Chem. 2022 May 5;18:497–507. doi: 10.3762/bjoc.18.52 (PMC9086497; doi:10.3762/bjoc.18.52)
Supplement: File 1 — Copies of NMR spectra and extended photophysical properties. [file Beilstein_J_Org_Chem-18-497-s001.pdf]

BEILSTEIN JOURNAL OF ORGANIC CHEMISTRY

Supporting Information

for

Substituent effect on TADF properties of 2-modified 4,6-bis(3,6-di-*tert*-butyl-9-carbazolyl)-5-methylpyrimidines

Irina Fiodorova, Tomas Serevičius, Rokas Skaisgiris, Saulius Juršėnas and Sigitas Tumkevicius

Beilstein J. Org. Chem. **2022,** *18,* 497–507. doi:10.3762/bjoc.18.52

Copies of NMR spectra and extended photophysical properties

License and Terms: This is a supporting information file under the terms of the Creative Commons Attribution License (https://creativecommons.org/licenses/by/4.0). Please note that the reuse, redistribution and reproduction in particular requires that the author(s) and source are credited and that individual graphics may be subject to special legal provisions.
The license is subject to the *Beilstein Journal of Organic Chemistry* terms and conditions: (https://www.beilstein-journals.org/bjoc/terms)

Table of contents

Copies of ^{1}H and ^{13}C NMR, and HRMS spectra S2
Extended photophysical properties S16

Copies of ^{1}H and ^{13}C NMR, and HRMS spectra

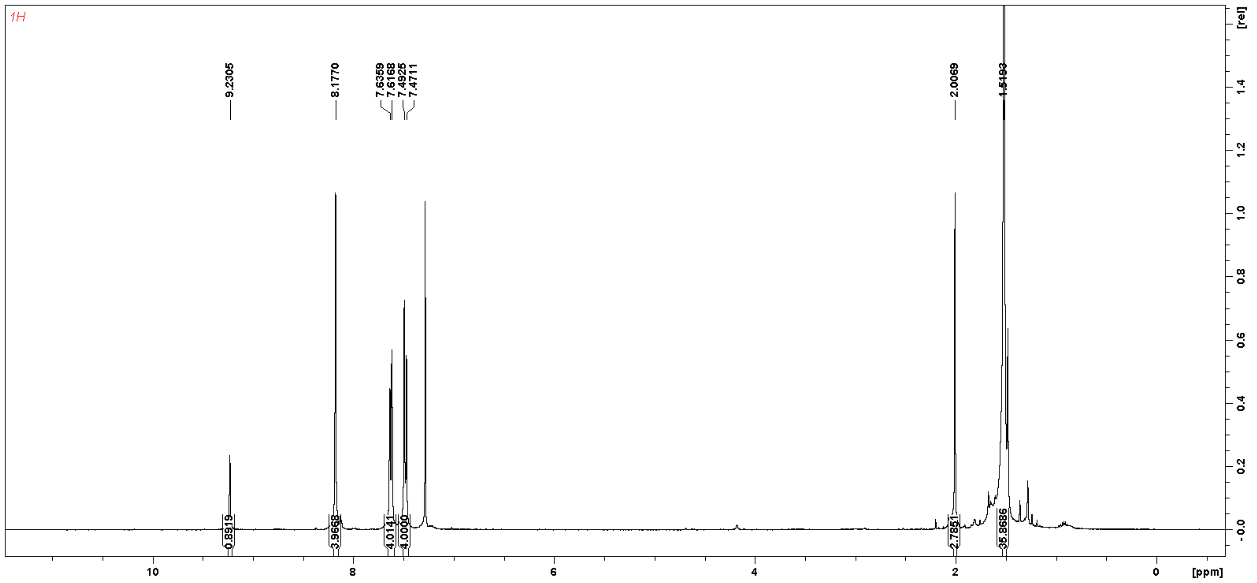

Figure S1. ^{1}H NMR spectrum of 4,6-bis[3,6-di(*tert*-butyl)-9*H*-carbazol-9-yl]-5-methylpyrimidine (**1**).

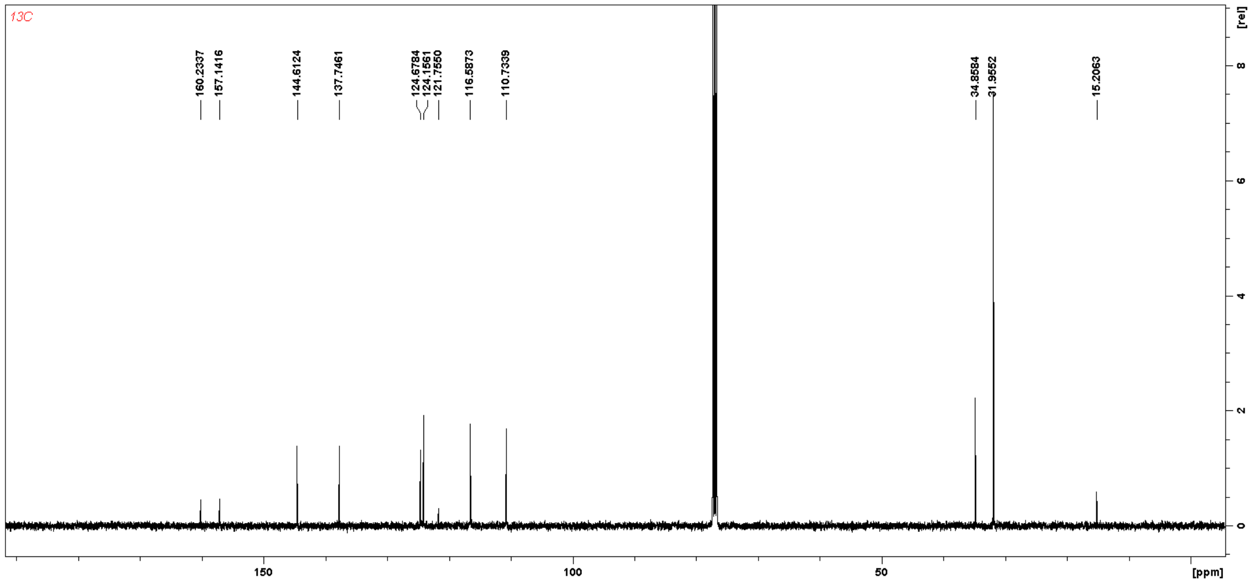

Figure S2. ^{13}C NMR spectrum of 4,6-bis[3,6-di(*tert*-butyl)-9*H*-carbazol-9-yl]-5-methylpyrimidine (**1**).

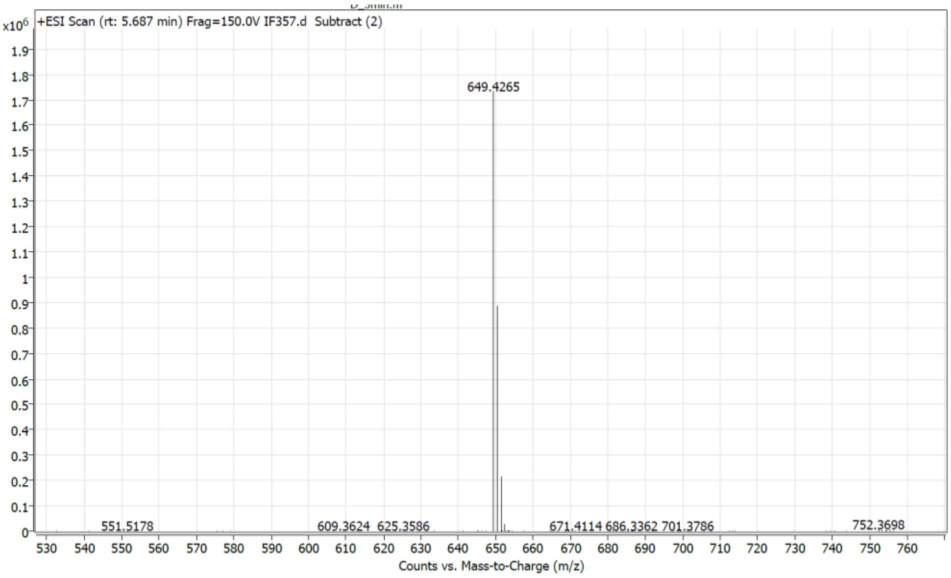

Figure S3. HRMS spectrum of 4,6-bis[3,6-di(*tert*-butyl)-9*H*-carbazol-9-yl]-5-methylpyrimidine (**1**).

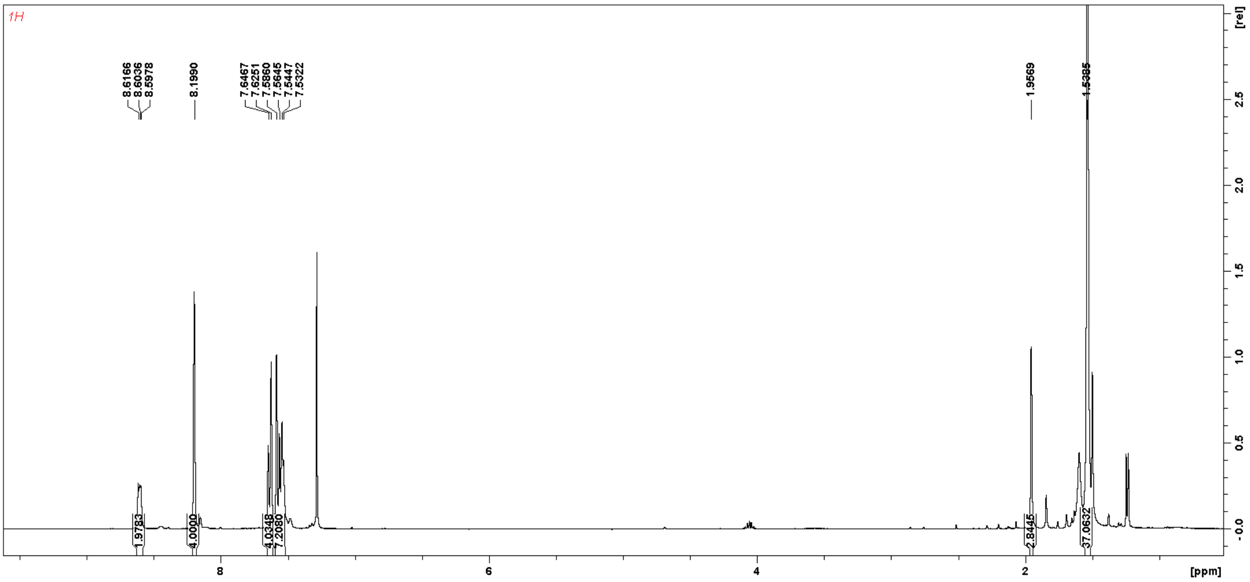

Figure S4. ^{1}H NMR spectrum of 4,6-bis[3,6-di(*tert*-butyl)-9*H*-carbazol-9-yl]-5-methyl-2-phenylpyrimidine (**2a**).

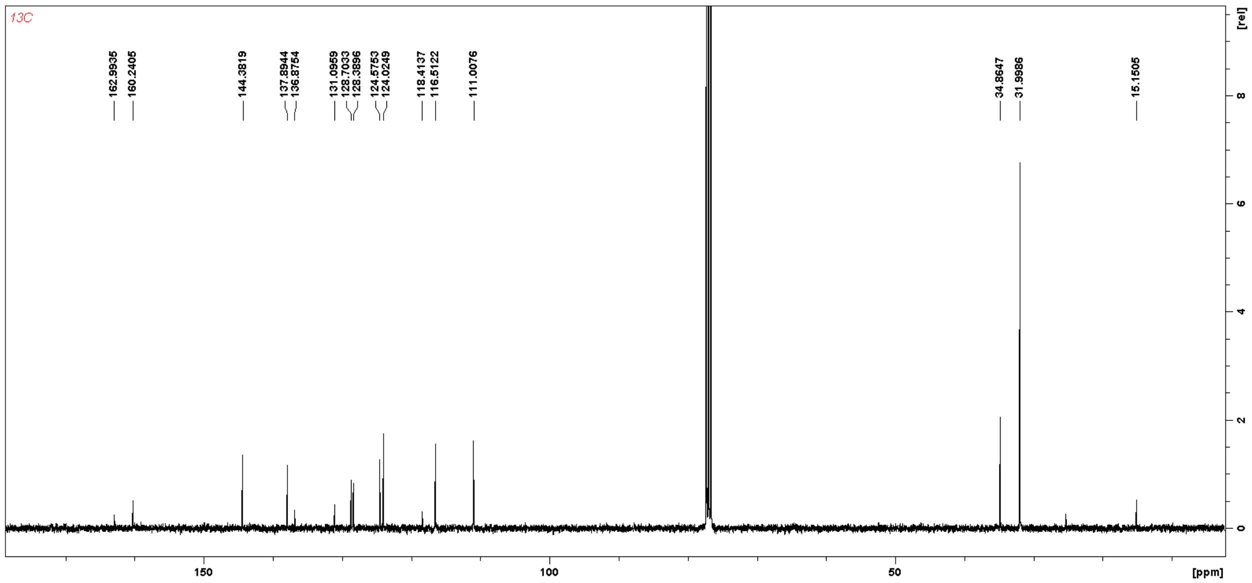

Figure S5.^{3}C NMR spectrum of 4,6-bis[3,6-di(*tert*-butyl)-9*H*-carbazol-9-yl]-5-methyl-2-phenylpyrimidine (**2a**).

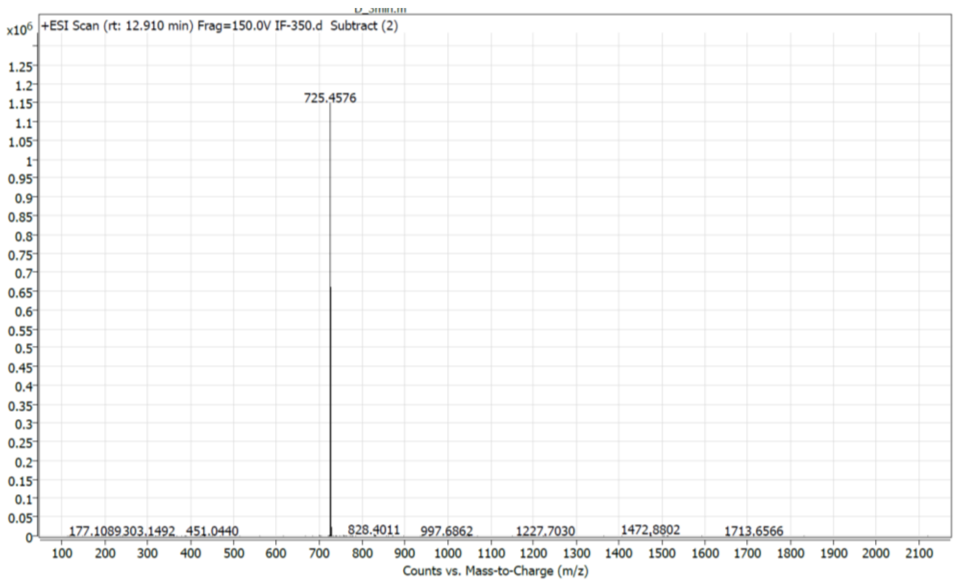

Figure S6. HRMS spectrum of 4,6-bis[3,6-di(*tert*-butyl)-9*H*-carbazol-9-yl]-5-methyl-2-phenylpyrimidine (**2a**).

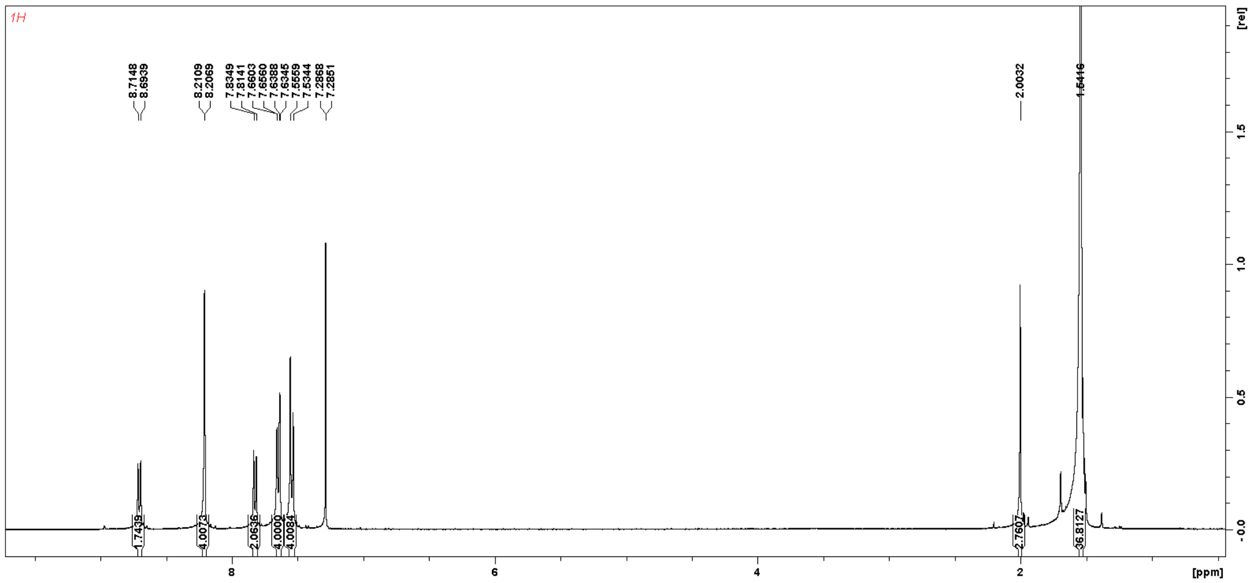

Figure S7. ^{1}H NMR spectrum of 4,6-bis[3,6-di(*tert*-butyl)-9*H*-carbazol-9-yl]-2-(4-cyanophenyl)-5-methylpyrimidine (**2b**).

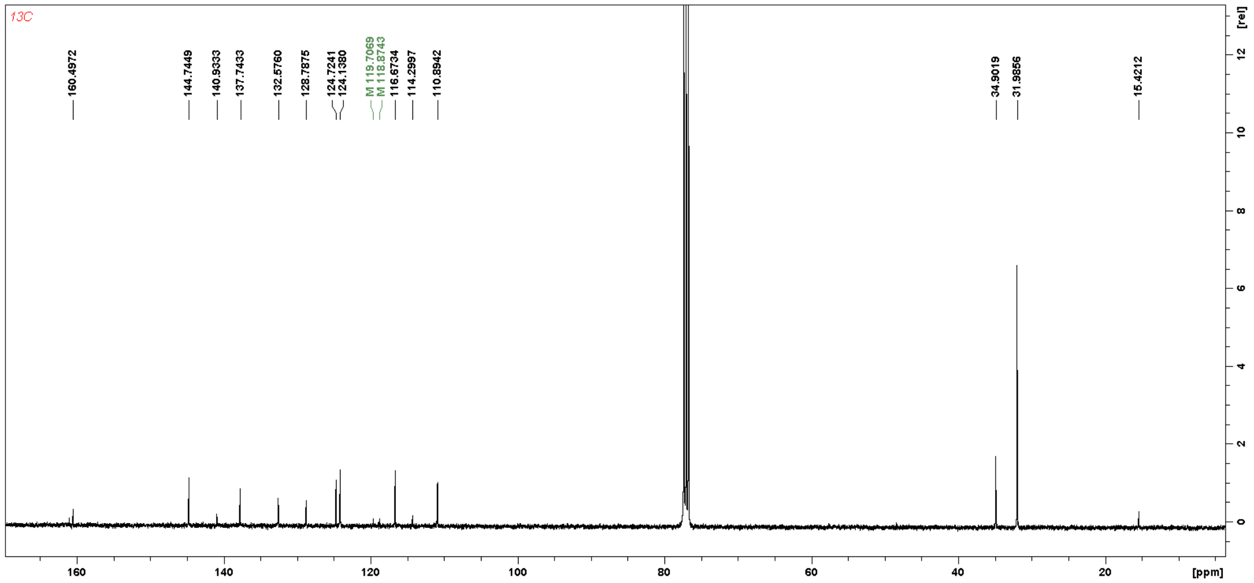

Figure S8. ^{13}C NMR spectrum of 4,6-bis[3,6-di(*tert*-butyl)-9*H*-carbazol-9-yl]-2-(4-cyanophenyl)-5-methylpyrimidine (**2b**).

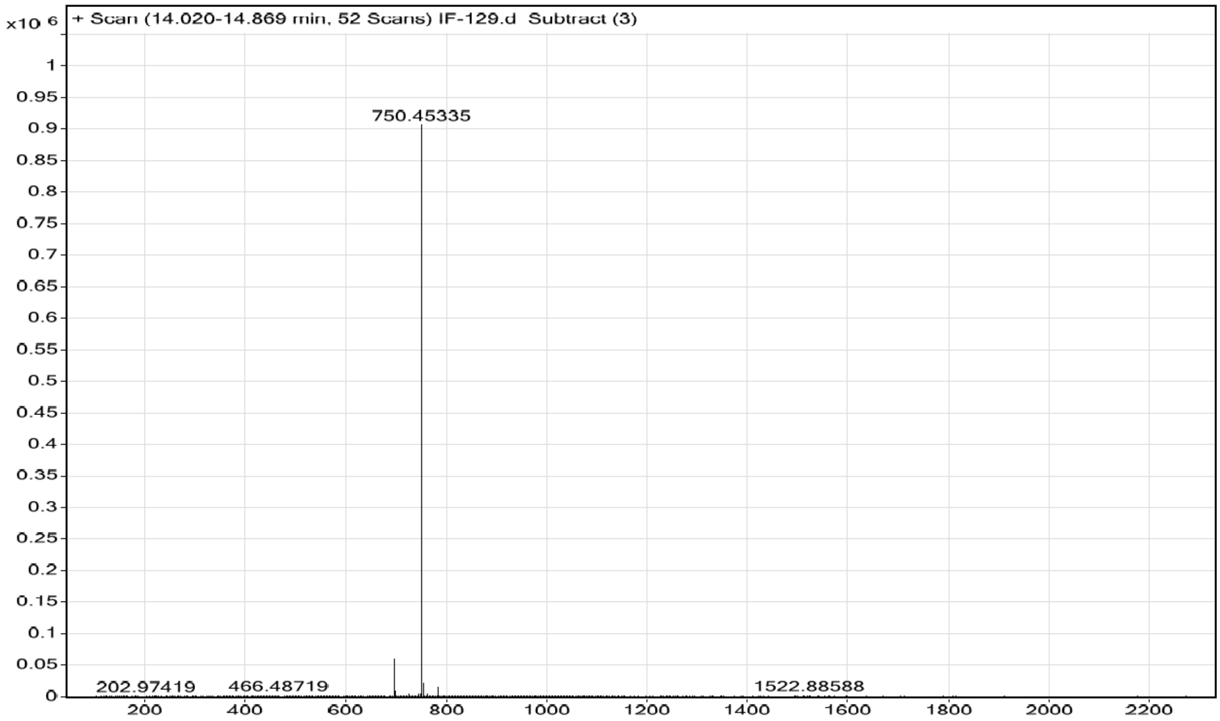

Figure S9. HRMS spectrum of 4,6-bis[3,6-di(*tert*-butyl)-9*H*-carbazol-9-yl]-2-(4-cyanophenyl)-5-methylpyrimidine (**2b**).

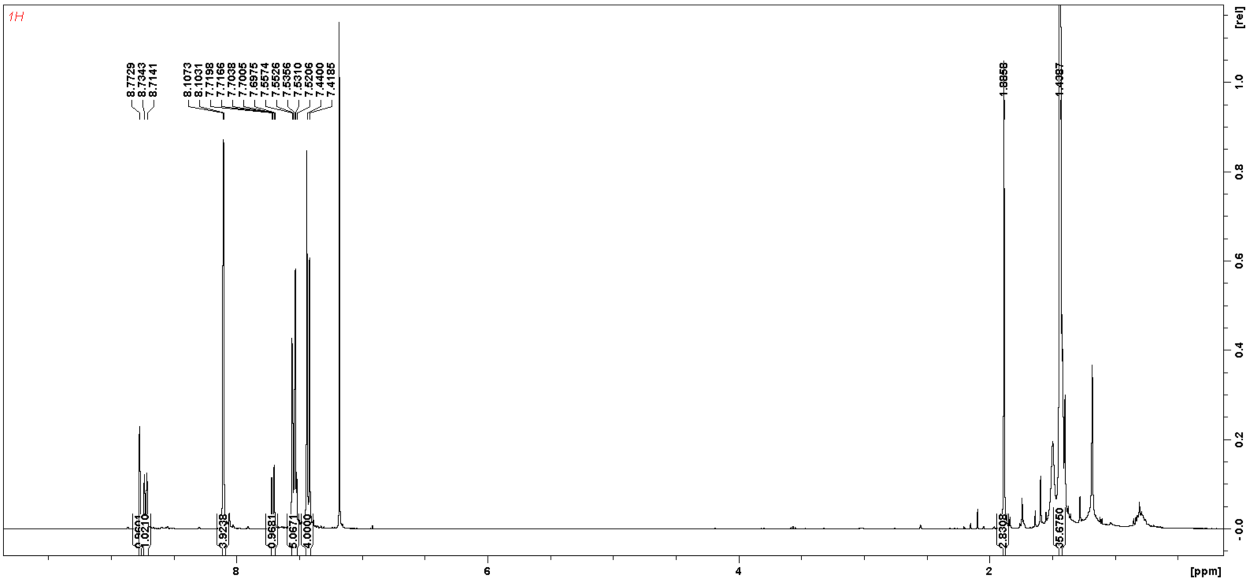

Figure S10. ^{1}H NMR spectrum of 4,6-bis[3,6-di(*tert*-butyl)-9*H*-carbazol-9-yl]-2-(3-cyanophenyl)-5-methylpyrimidine (**2c**).

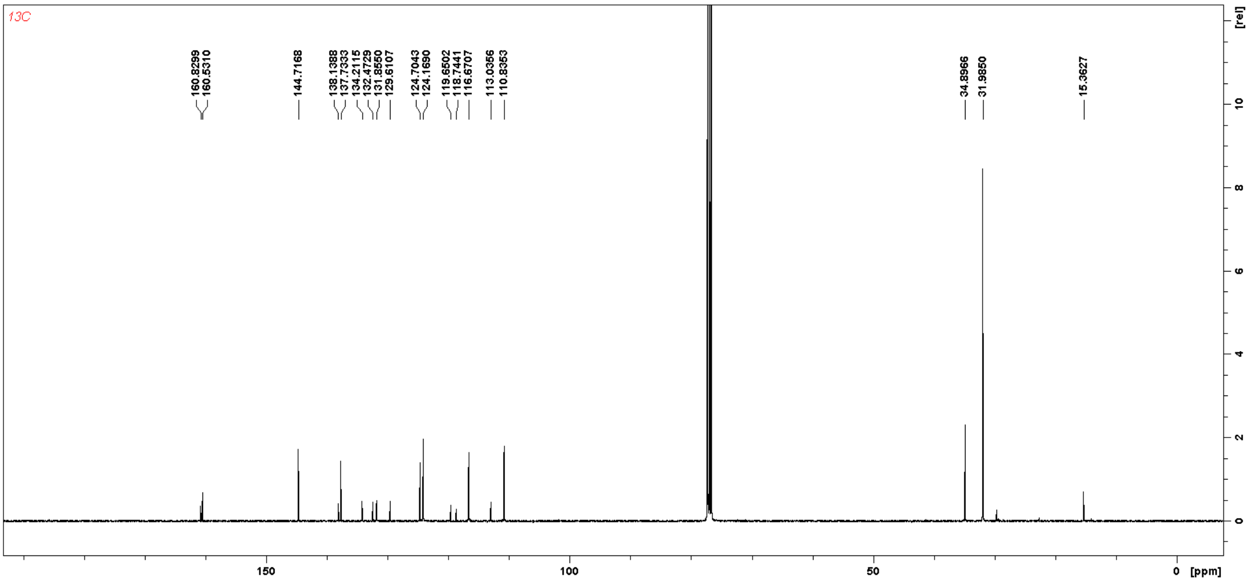

Figure S11. ^{13}C NMR spectrum of 4,6-bis[3,6-di(*tert*-butyl)-9*H*-carbazol-9-yl]-2-(3-cyanophenyl)-5-methylpyrimidine (**2c**).

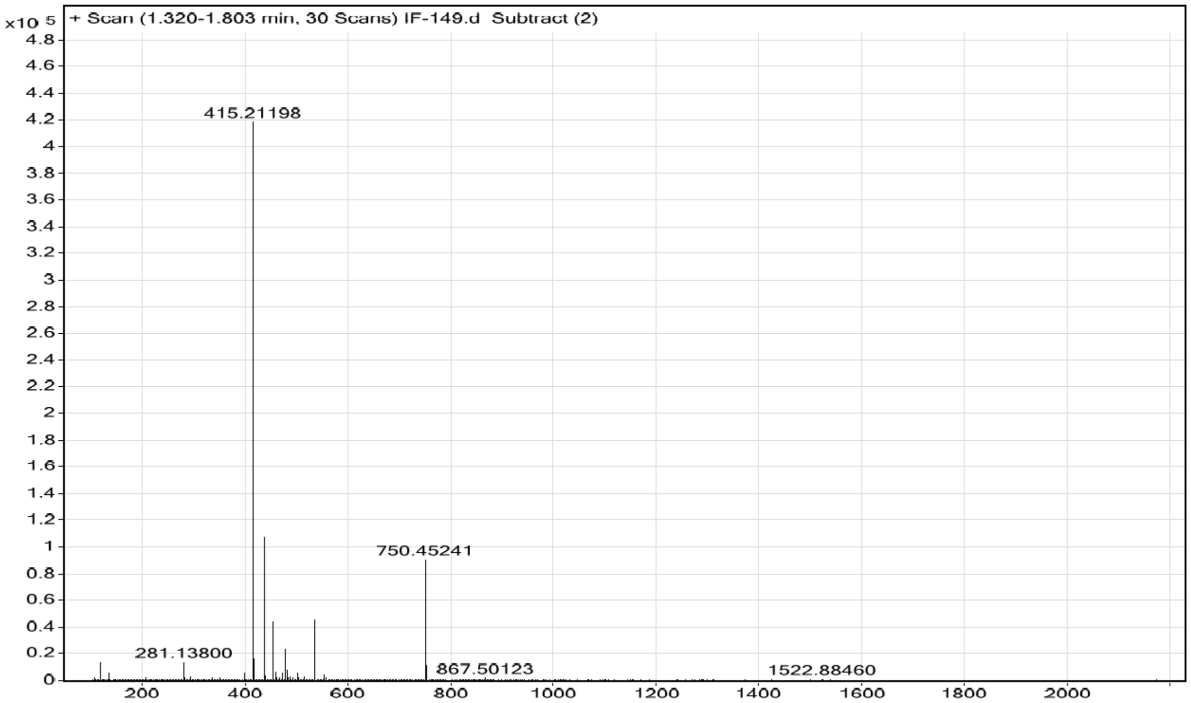

Figure S12. HRMS spectrum of 4,6-bis[3,6-di(*tert*-butyl)-9*H*-carbazol-9-yl]-2-(3-cyanophenyl)-5-methylpyrimidine (**2c**).

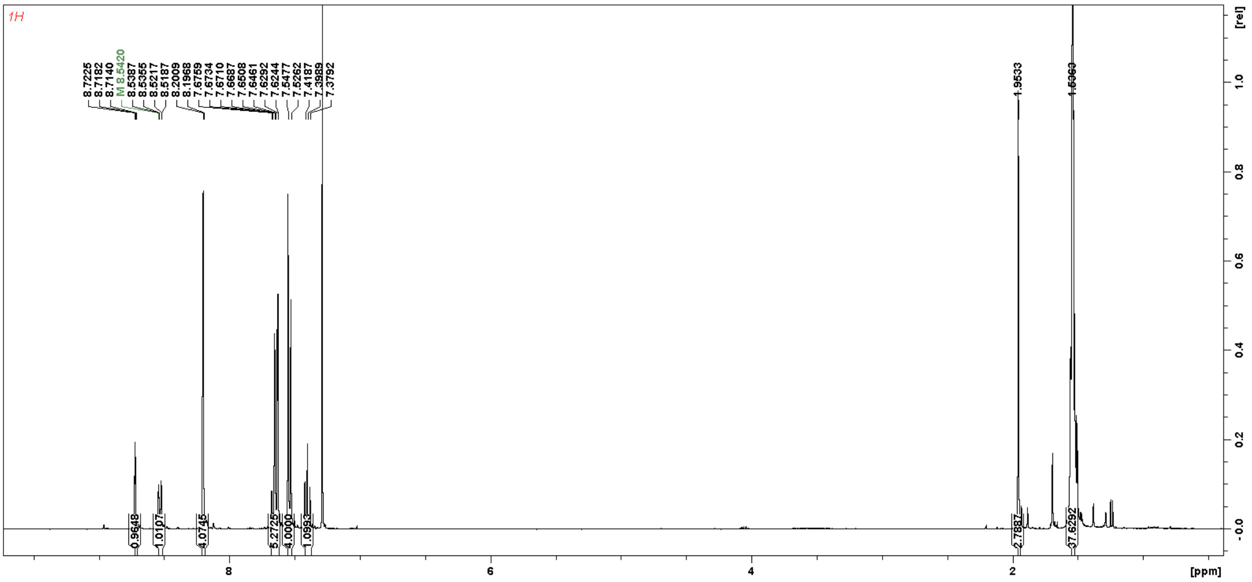

Figure S13. ^{1}H NMR spectrum of 4,6-bis[3,6-di(*tert*-butyl)-9*H*-carbazol-9-yl]-2-(3-bromophenyl)-5-methylpyrimidine (**2d**).

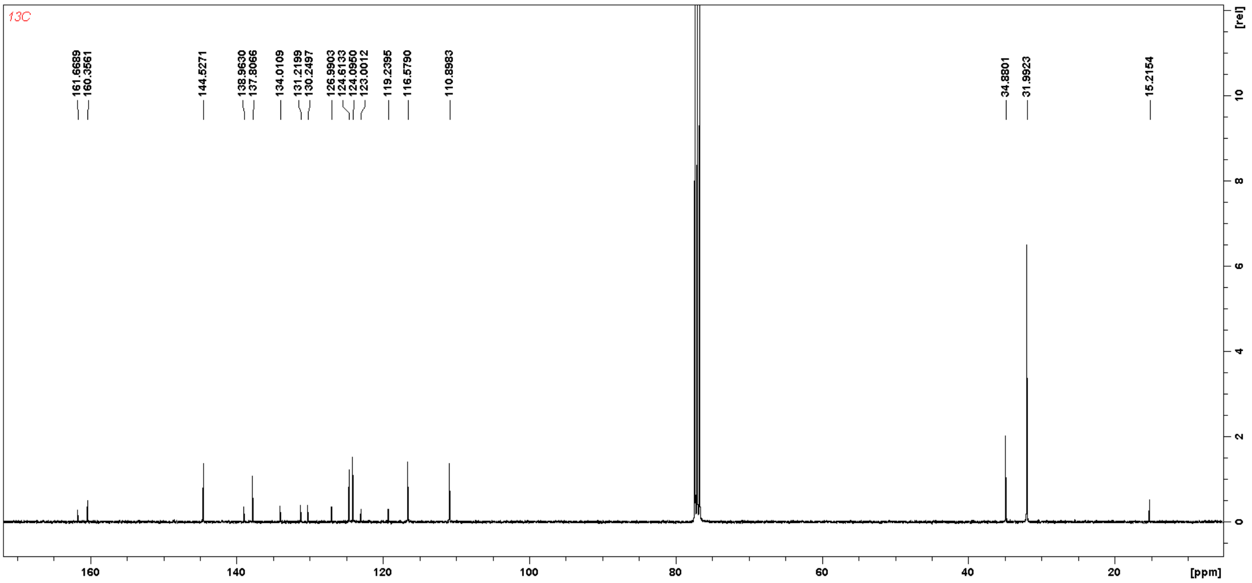

Figure S14. ^{13}C NMR spectrum of 4,6-bis[3,6-di(*tert*-butyl)-9*H*-carbazol-9-yl]-2-(3-bromophenyl)-5-methylpyrimidine (**2d**).

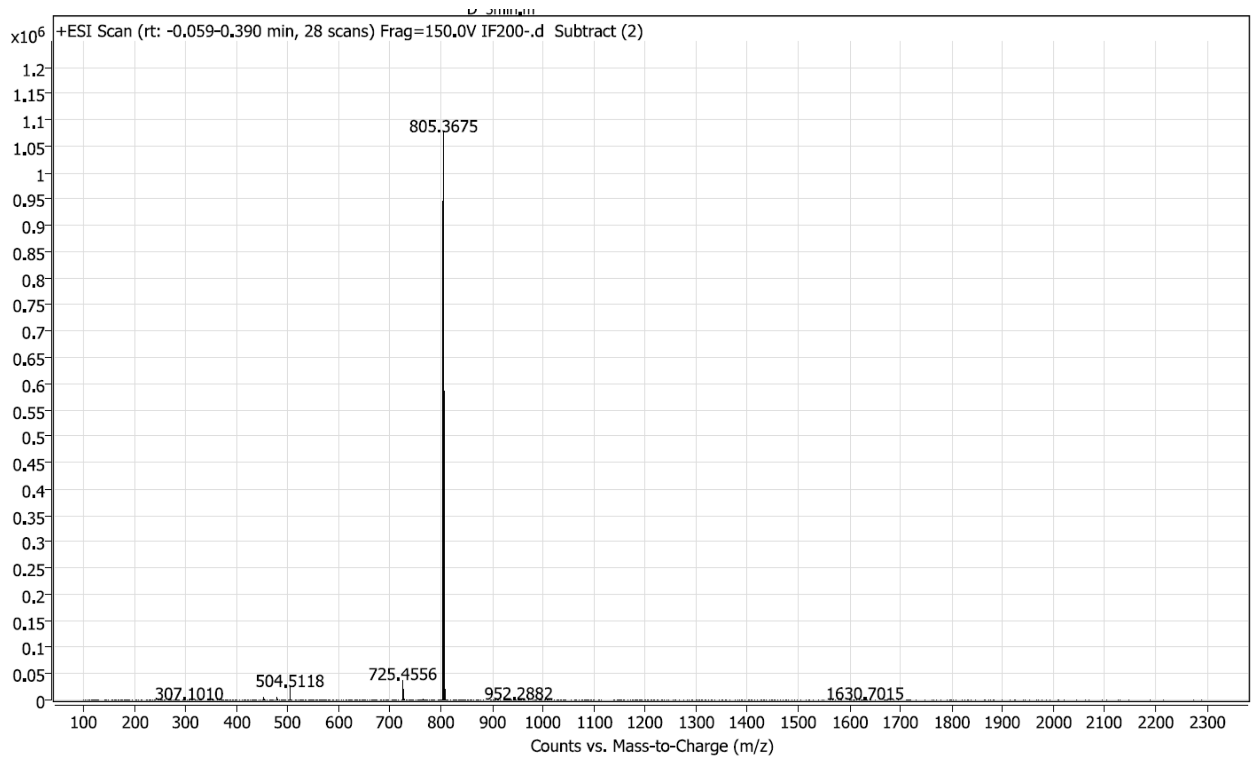

Figure S15. HRMS spectrum of 4,6-bis[3,6-di(*tert*-butyl)-9*H*-carbazol-9-yl]-2-(3-bromophenyl)-5-methylpyrimidine (**2d**).

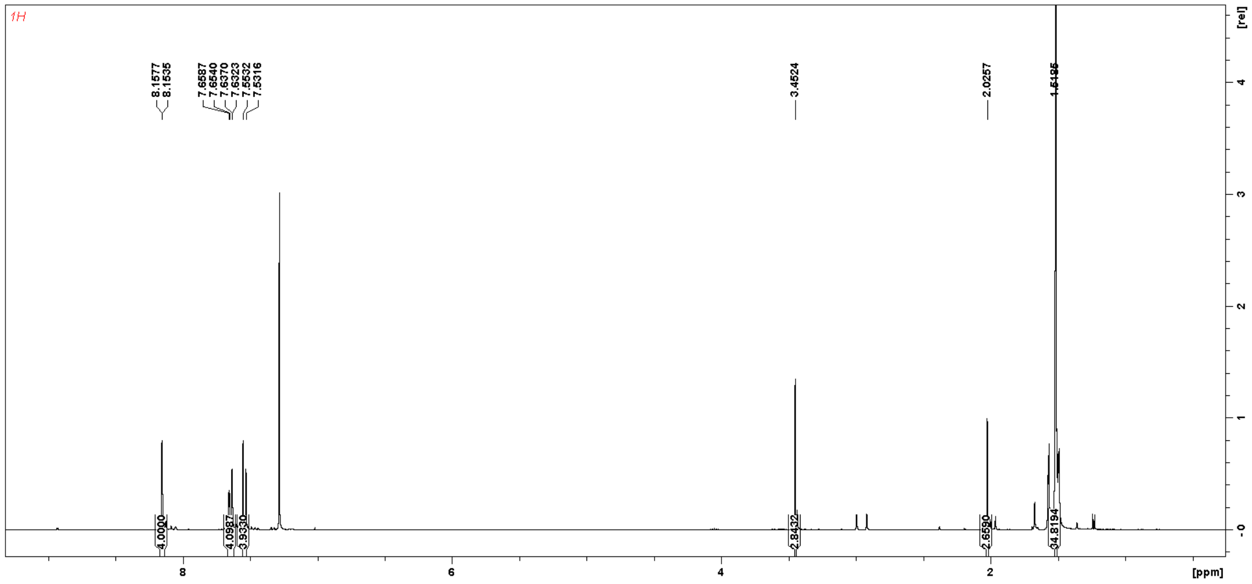

Figure S16. ^{1}H NMR spectrum of 4,6-bis(3,6-di-*tert*-butyl-9*H*-carbazol-9-yl)-5-methyl-2-methylsulfonylpyrimidine (**3**).

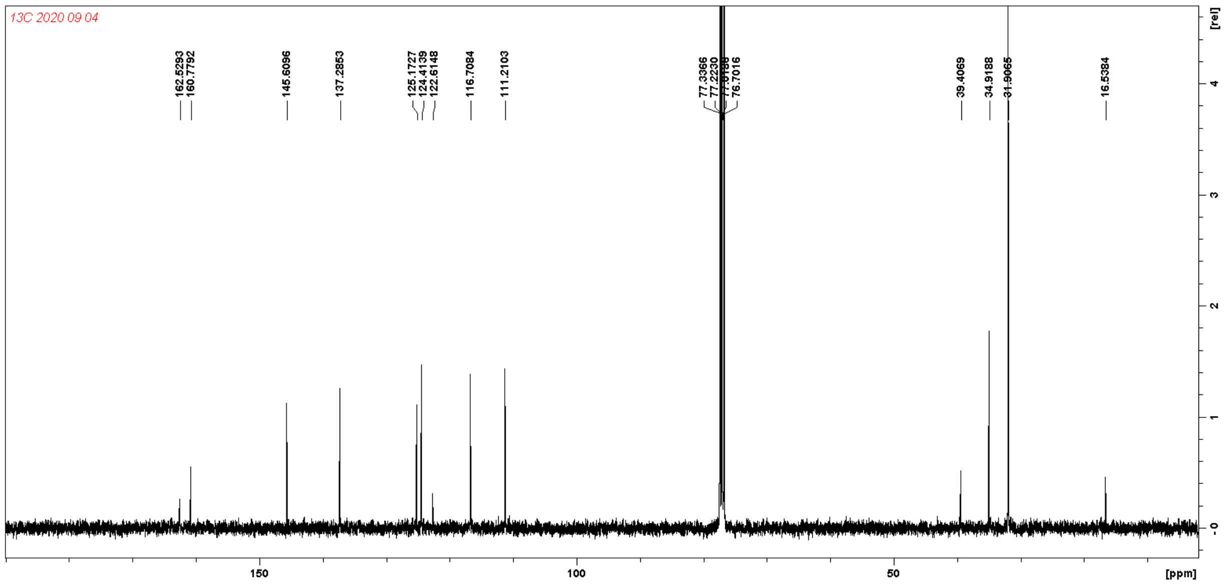

Figure S17. ^{13}C NMR spectrum of 4,6-bis(3,6-di-*tert*-butyl-9*H*-carbazol-9-yl)-5-methyl-2-methylsulfonylpyrimidine (**3**).

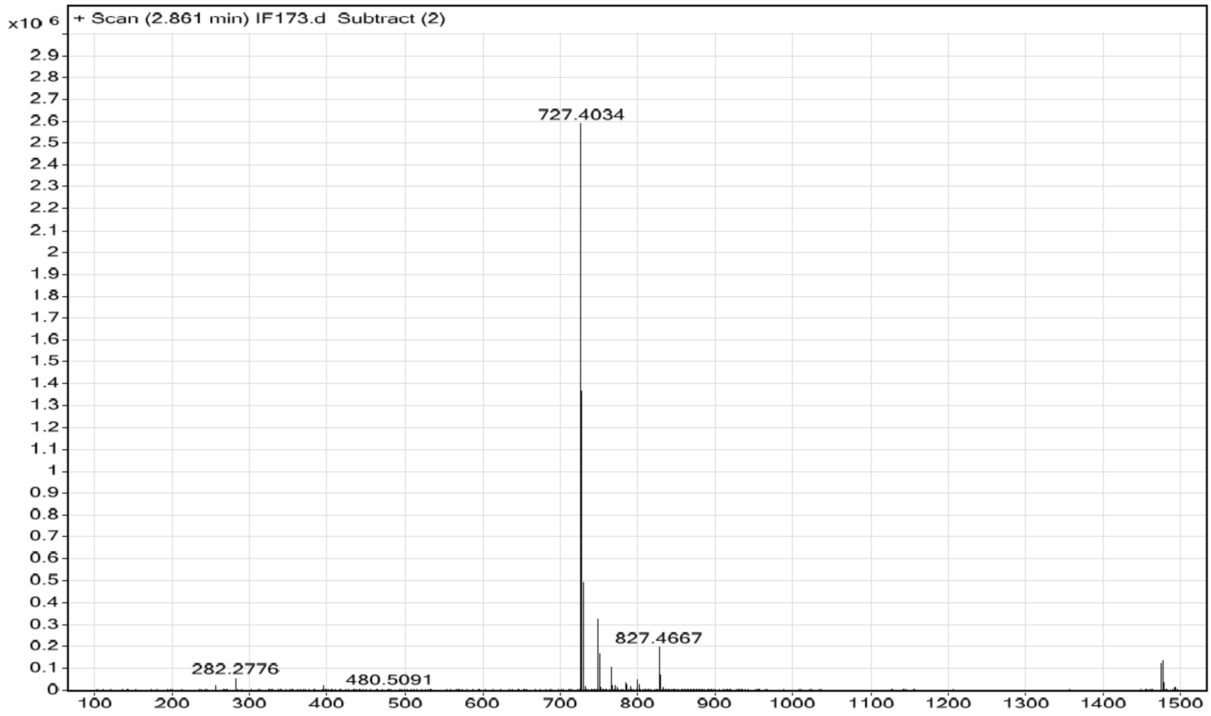

Figure S18. HRMS spectrum of 4,6-bis(3,6-di-*tert*-butyl-9*H*-carbazol-9-yl)-5-methyl-2-methylsulfonylpyrimidine (**3**).

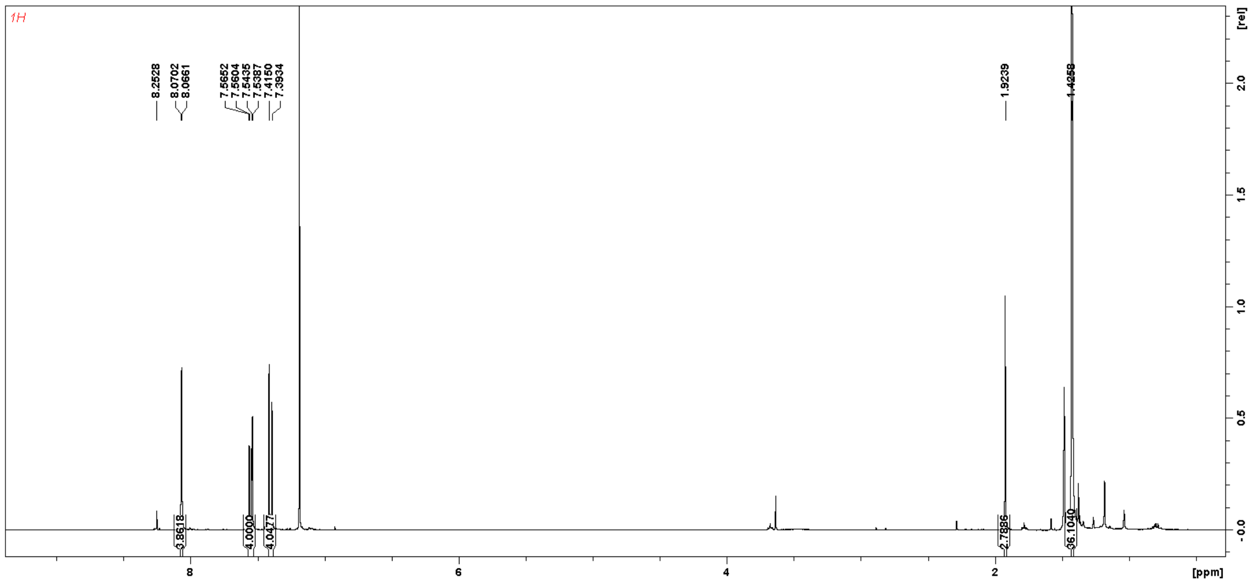

Figure S19. ^{1}H NMR spectrum of 4,6-bis(3,6-di-*tert*-butyl-9*H*-carbazol-9-yl)-5-methylpyrimidine-2-carbonitrile (**4**).

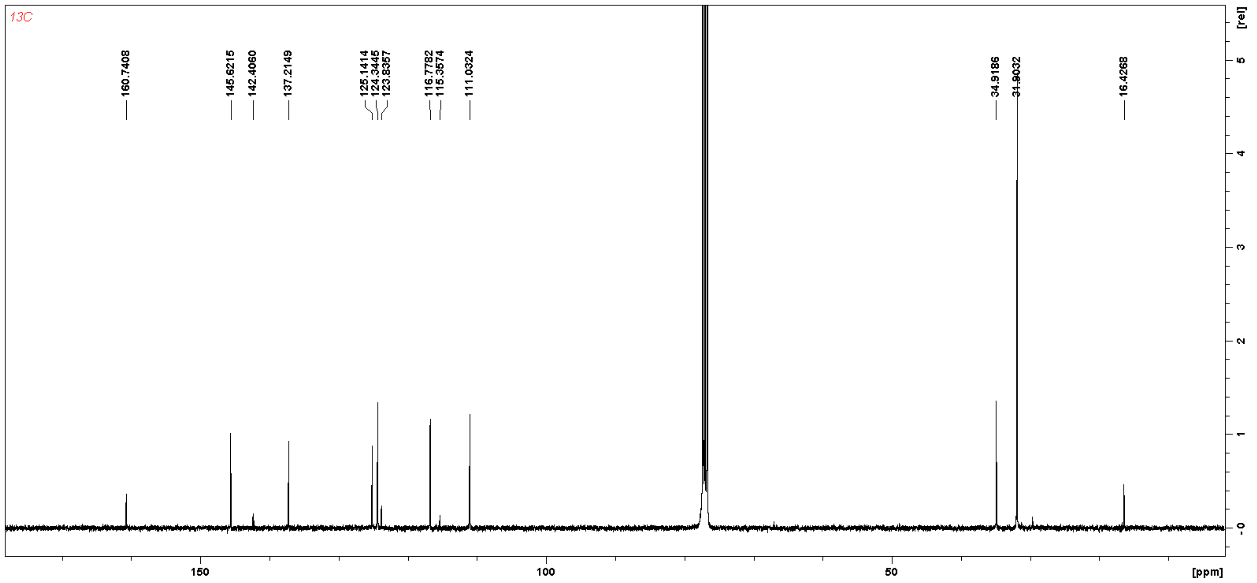

Figure S20. ^{13}C NMR spectrum of 4,6-bis(3,6-di-*tert*-butyl-9*H*-carbazol-9-yl)-5-methylpyrimidine-2-carbonitrile (**4**).

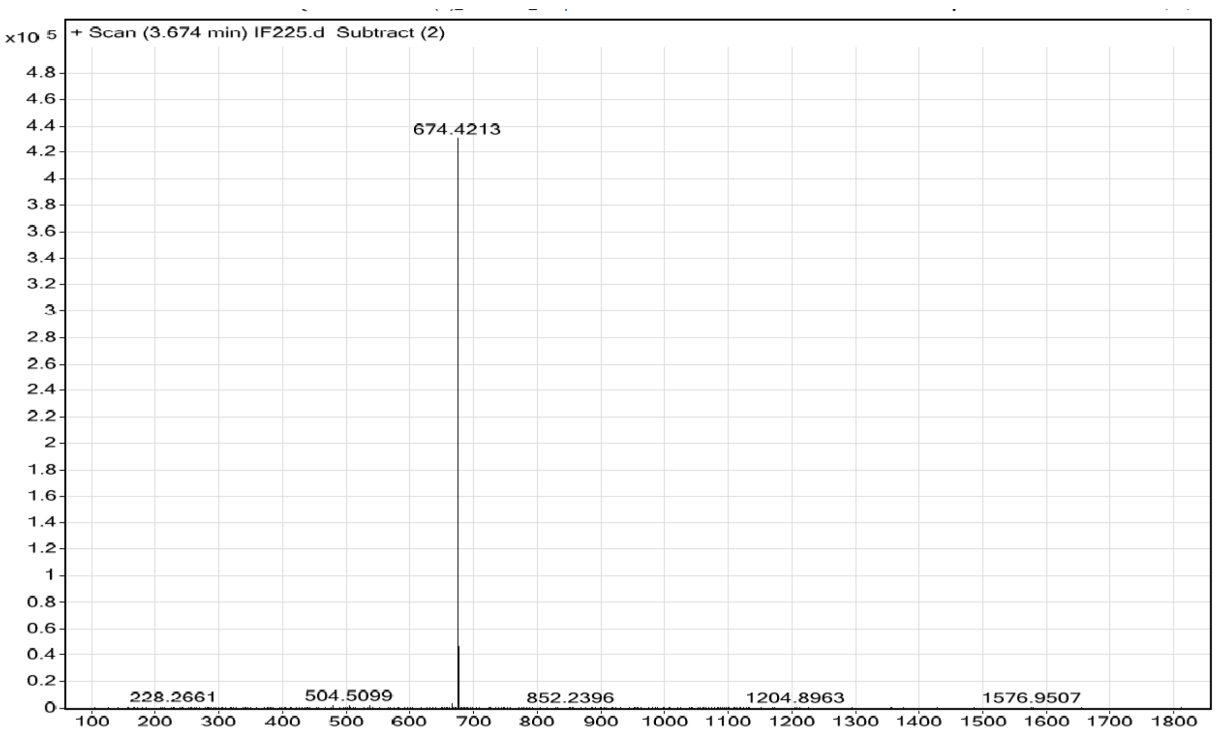

Figure S21. HRMS spectrum of 4,6-bis(3,6-di-*tert*-butyl-9*H*-carbazol-9-yl)-5-methylpyrimidine-2-carbonitrile (**4**).

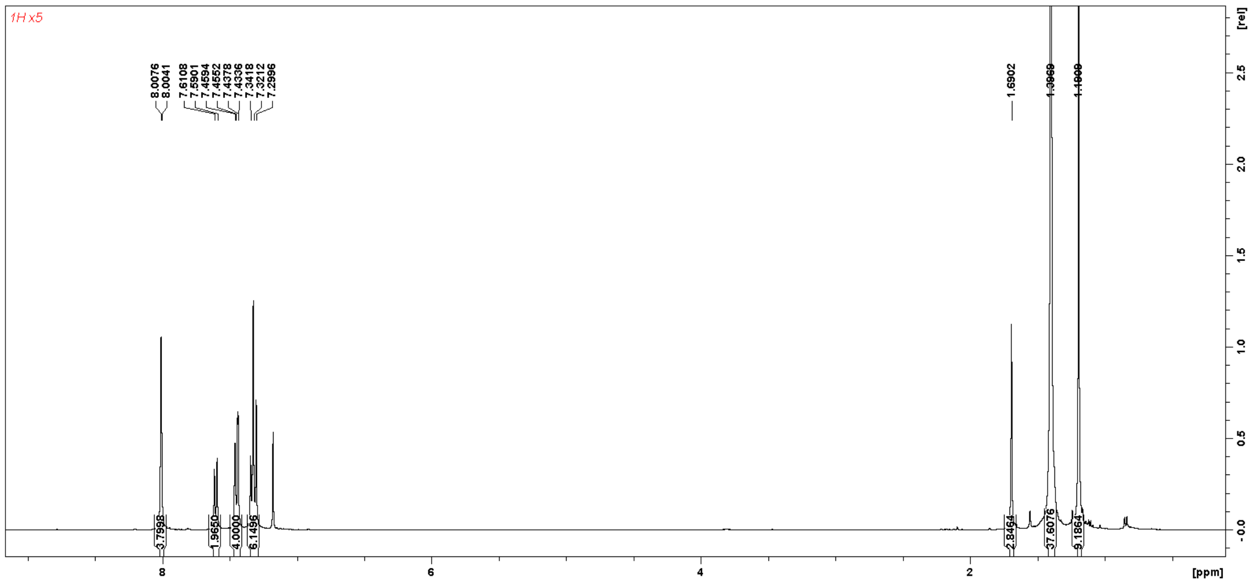

Figure S22. ^{1}H NMR spectrum of 4,6-bis[3,6-di(*tert*-butyl)-9*H*-carbazol-9-yl]-2-[4-(*tert*-butyl)phenylthio]-5-methylpyrimidine (**5**).

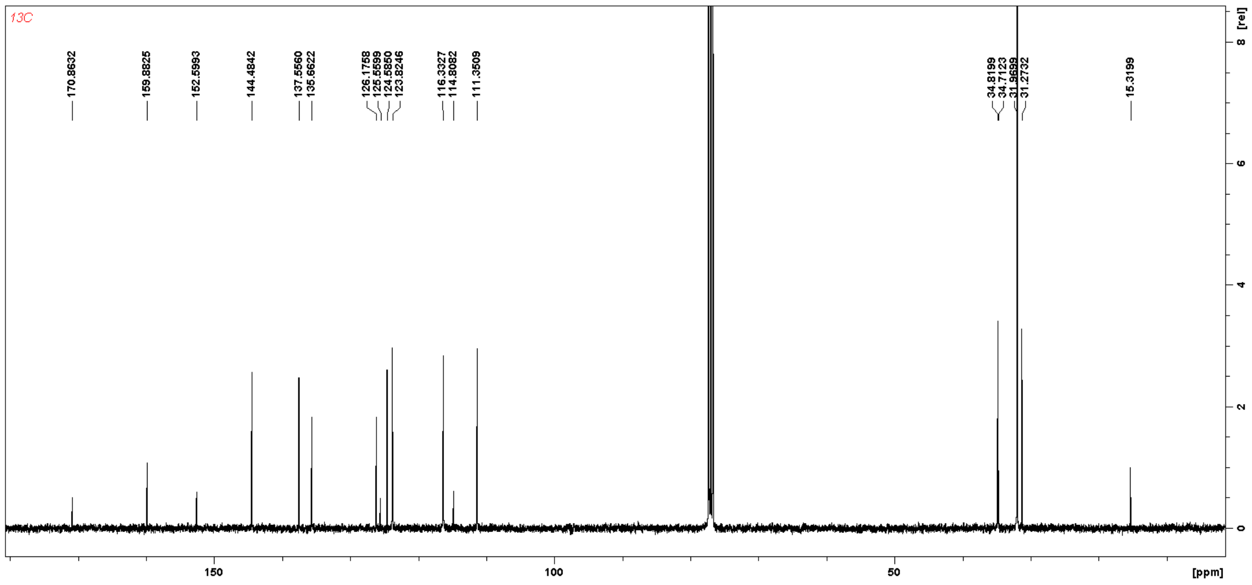

Figure S23. ^{13}C NMR spectrum of 4,6-bis[3,6-di(*tert*-butyl)-9*H*-carbazol-9-yl]-2-[4-(*tert*-butyl)phenylthio]-5-methylpyrimidine (**5**).

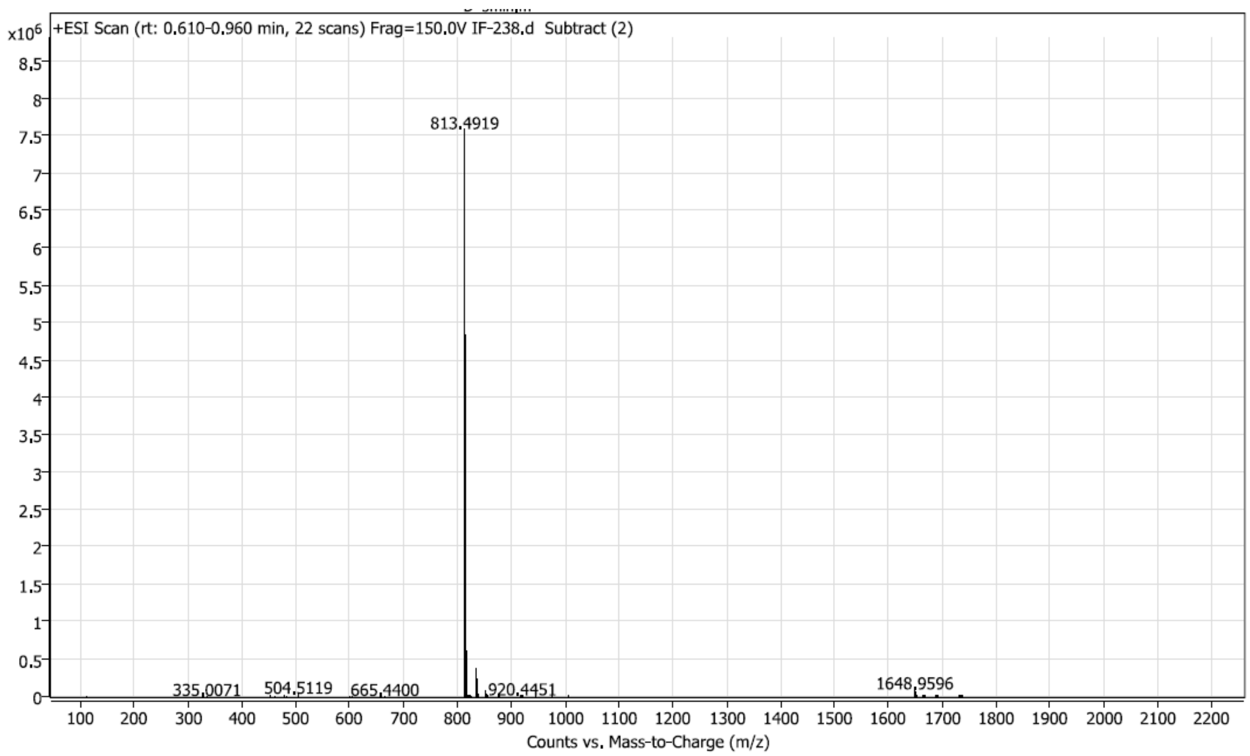

Figure S24. HRMS spectrum of 4,6-bis[3,6-di(*tert*-butyl)-9*H*-carbazol-9-yl]-2-[4-(*tert*-butyl)phenylthio]-5-methylpyrimidine (**5**).

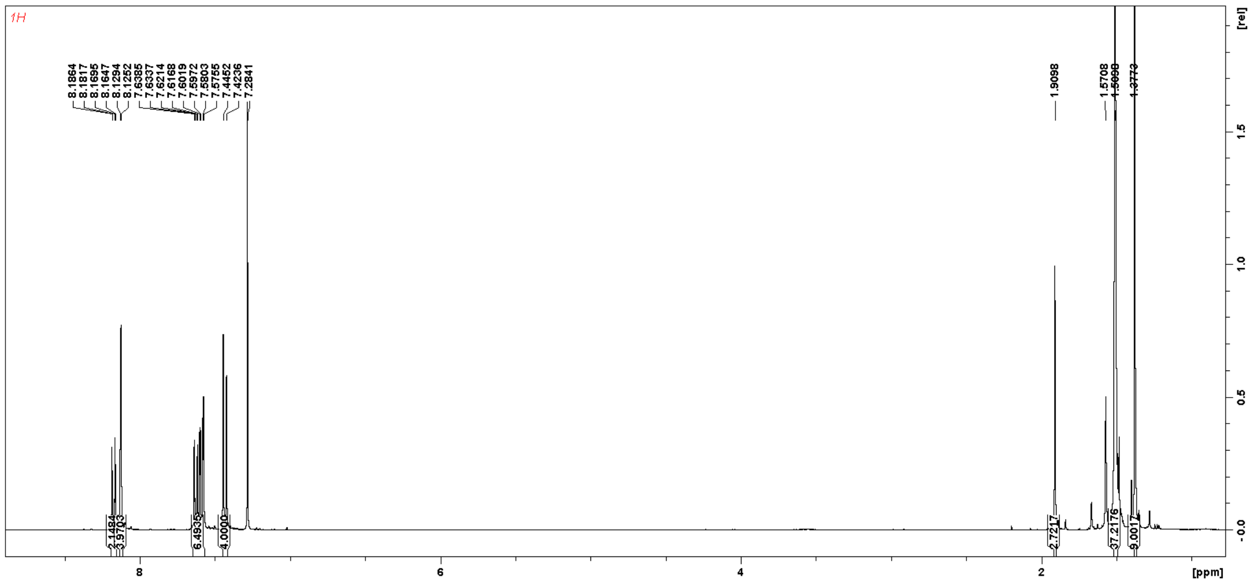

Figure S25. ^{1}H NMR spectrum of 4,6-bis[3,6-di(*tert*-butyl)-9*H*-carbazol-9-yl]-2-[4-(*tert*-butyl)phenylsulfonyl]-5-methylpyrimidine (**6**).

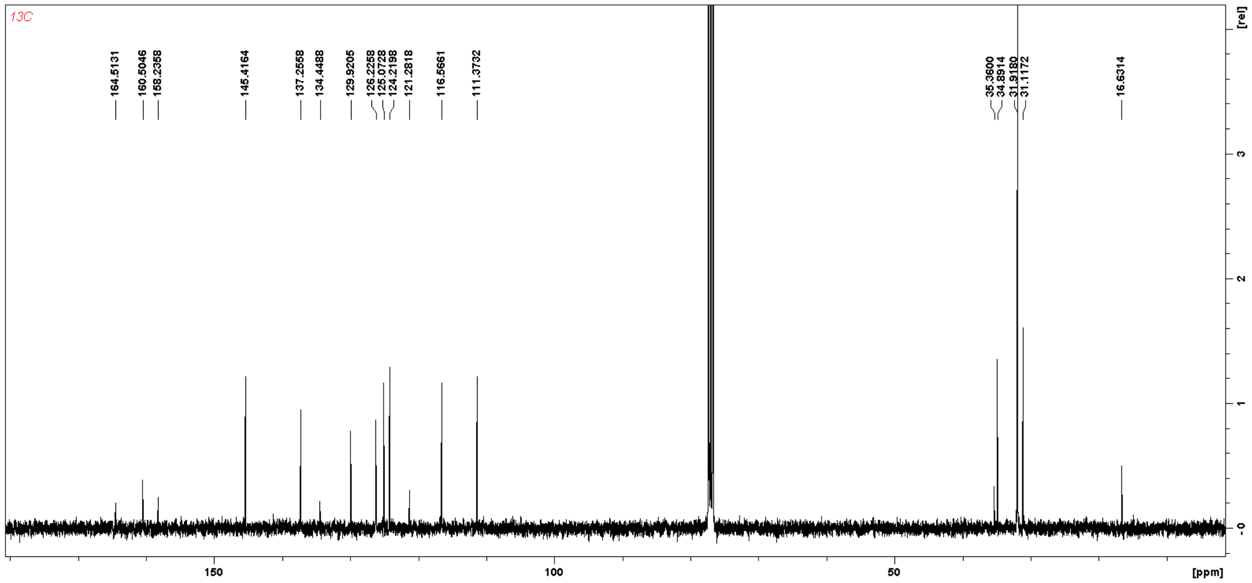

Figure S26. ^{13}C NMR spectrum of 4,6-bis[3,6-di(*tert*-butyl)-9*H*-carbazol-9-yl]-2-[4-(*tert*-butyl)phenylsulfonyl]-5-methylpyrimidine (**6**).

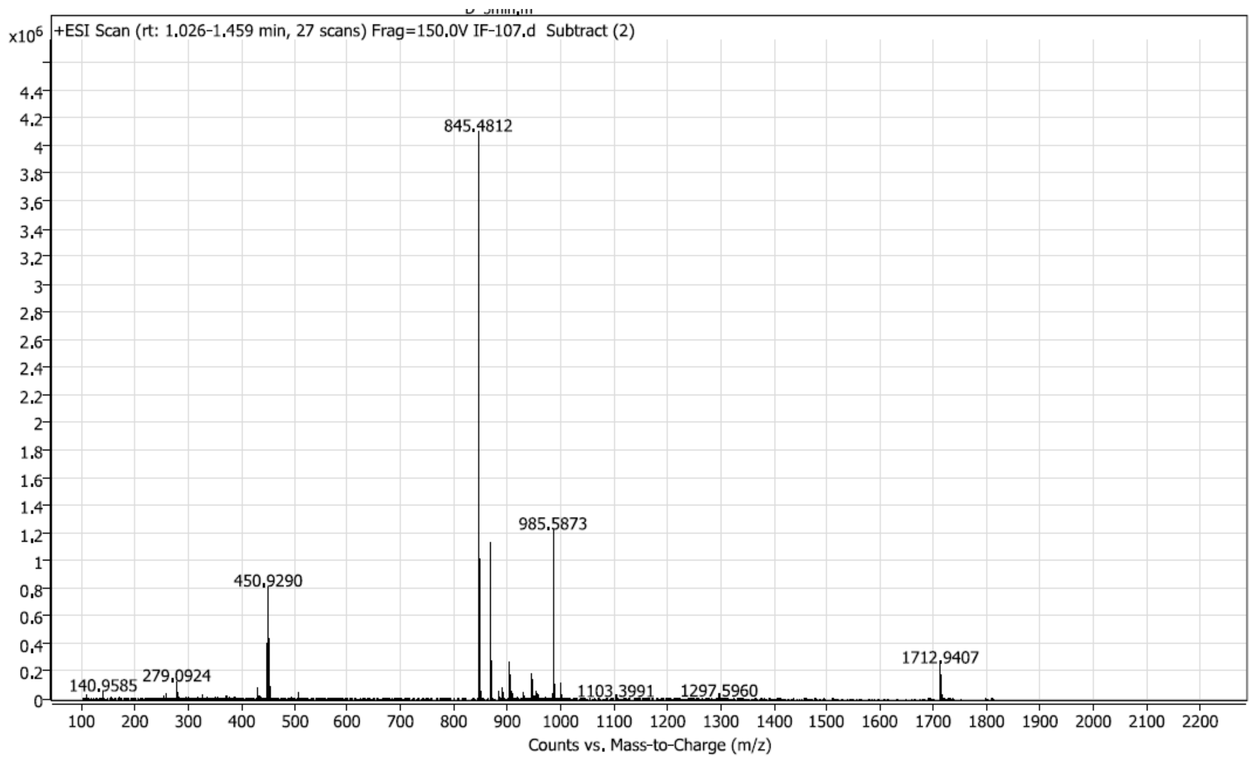

Figure S27. HRMS spectrum of 4,6-bis[3,6-di(*tert*-butyl)-9*H*-carbazol-9-yl]-2-[4-(*tert*-butyl)phenylsulfonyl]-5-methylpyrimidine (**6**).

Extended photophysical properties

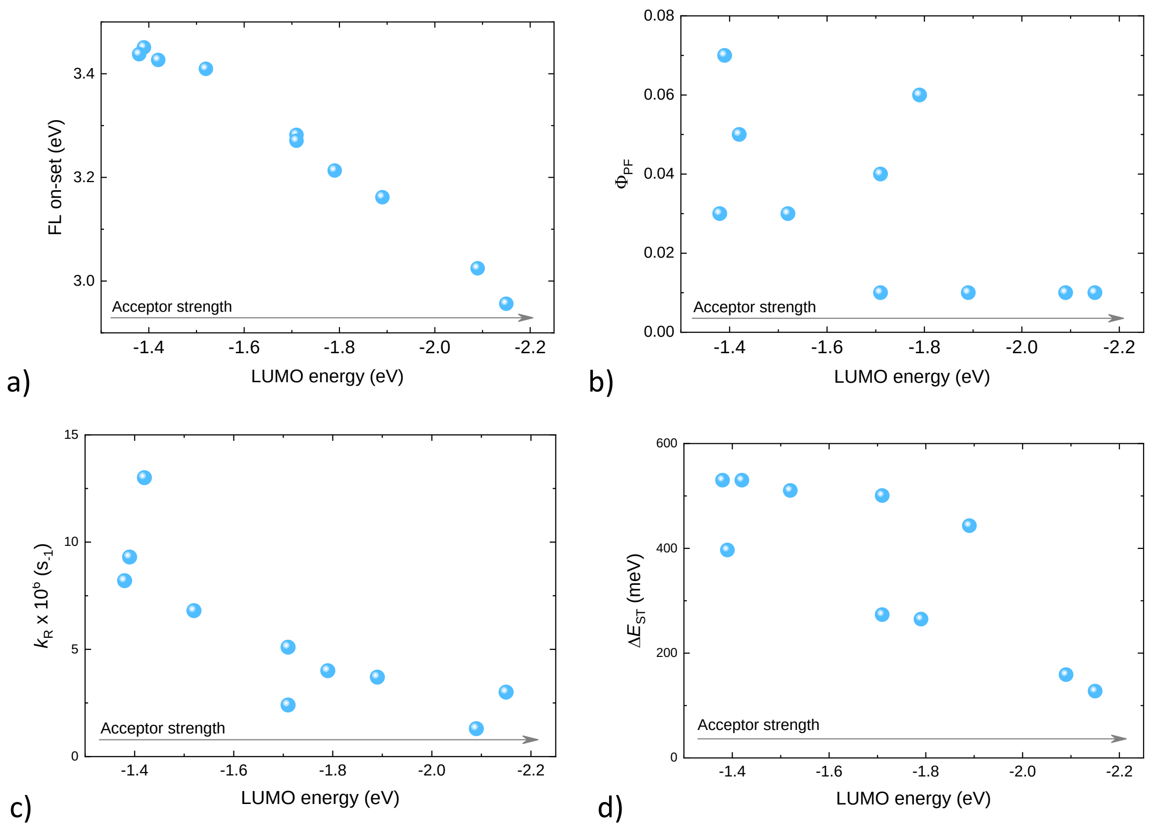

Figure S28. a) Fluorescence on-set energy, b) PF quantum yield, c) k_r and d) ΔE_{ST} as a function of LUMO energy.

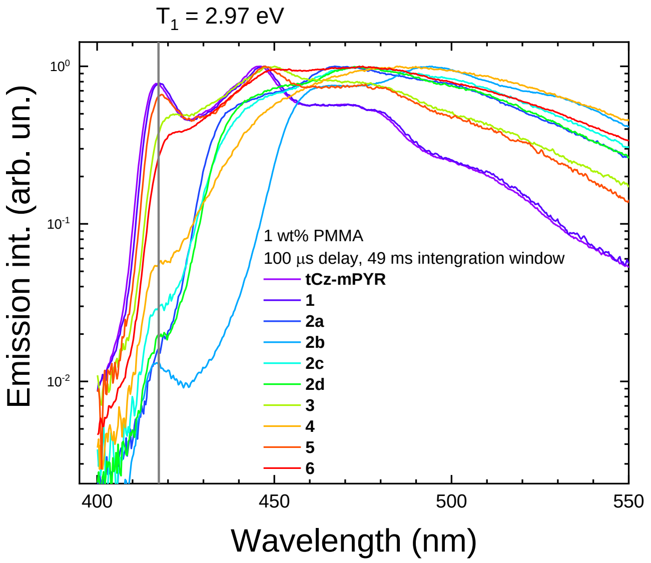

Figure S29. Phosphorescence spectra of carbazole–pyrimidine TADF compounds in semi-logarithmic scale.

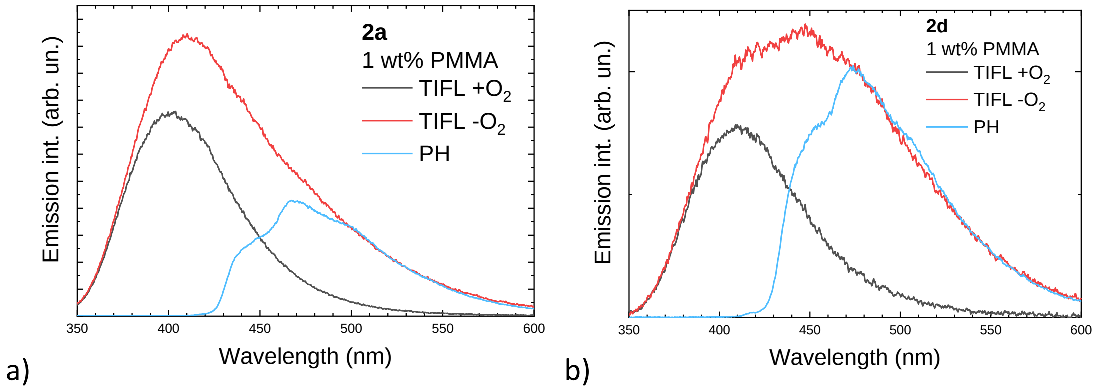

Figure S30. a) TIFL spectra of 1 wt % PMMA films of **2a** in oxygen-saturated (+O_2) and oxygen-free conditions (-O_2) together with PH spectrum at 10 K (intensity of PH spectrum was modified to overlay with low-energy shoulder of TIFL). b) TIFL spectra of 1 wt % PMMA films of **2d** in oxygen-saturated (+O_2) and oxygen-free conditions (-O_2) together with PH spectrum at 10 K (intensity of PH spectrum was modified to overlay with low-energy shoulder of TIFL).

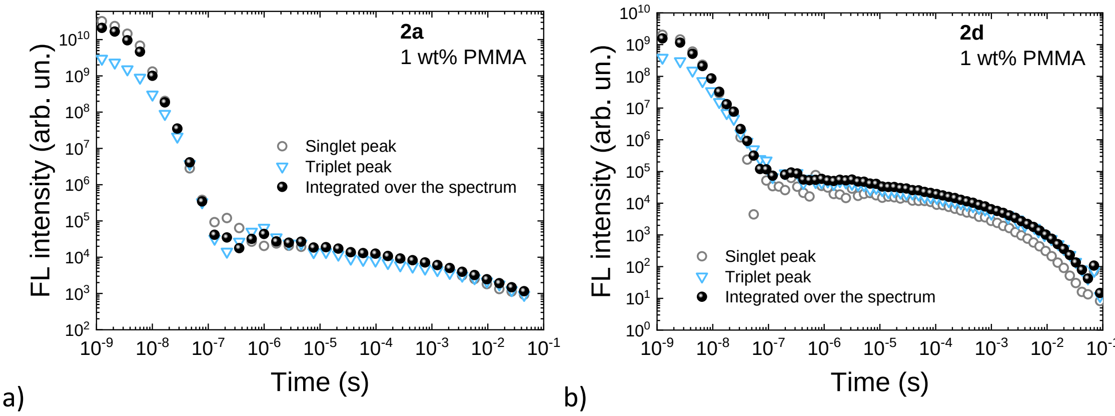

Figure S31. Fluorescence decay transients of 1 wt % PMMA films of **2a** and **2d** in oxygen-free conditions. Grey figures are transients at singlet emission peak, blue figures are transients at triplet emission peak and black figures are the sum of both decays.
